# Unprecedented enhancement of recombinant protein production in sugarcane culms using a combinatorial promoter stacking system

**DOI:** 10.1038/s41598-020-70530-z

**Published:** 2020-08-13

**Authors:** Mona B. Damaj, John L. Jifon, Susan L. Woodard, Carol Vargas-Bautista, Georgia O. F. Barros, Joe Molina, Steven G. White, Bassam B. Damaj, Zivko L. Nikolov, Kranthi K. Mandadi

**Affiliations:** 1 Texas A&M AgriLife Research and Extension Center, 2415 East US Highway 83, Weslaco, TX 78596 USA; 2 Department of Horticultural Sciences, Texas A&M University, College Station, TX 77843-2133 USA; 3 National Center for Therapeutics Manufacturing, Texas A&M University, 100 Discovery Drive, College Station, TX 77843-4482 USA; 4 BioSeparation Laboratory, Biological and Agricultural Engineering Department, College Station, TX 77843-2117 USA; 5 Innovus Pharmaceuticals, Inc., 8845 Rehco Road, San Diego, CA 92121 USA; 6 Department of Plant Pathology and Microbiology, Texas A&M University, College Station, TX 77843-2132 USA; 7 Present Address: College of Medicine, Texas A&M University, 8447 Riverside Parkway, Bryan, TX 77807 USA

**Keywords:** Expression systems, Biomaterials - proteins, Biofuels, Plant sciences

## Abstract

Plants represent a safe and cost-effective platform for producing high-value proteins with pharmaceutical properties; however, the ability to accumulate these in commercially viable quantities is challenging. Ideal crops to serve as biofactories would include low-input, fast-growing, high-biomass species such as sugarcane. The objective of this study was to develop an efficient expression system to enable large-scale production of high-value recombinant proteins in sugarcane culms. Bovine lysozyme (BvLz) is a potent broad-spectrum antimicrobial enzyme used in the food, cosmetics and agricultural industries. Here, we report a novel strategy to achieve high-level expression of recombinant proteins using a combinatorial stacked promoter system. We demonstrate this by co-expressing *BvLz* under the control of multiple constitutive and culm-regulated promoters on separate expression vectors and combinatorial plant transformation. BvLz accumulation reached 1.4% of total soluble protein (TSP) (10.0 mg BvLz/kg culm mass) in stacked multiple promoter:*BvLz* lines, compared to 0.07% of TSP (0.56 mg/kg) in single promoter:*BvLz* lines. BvLz accumulation was further boosted to 11.5% of TSP (82.5 mg/kg) through event stacking by re-transforming the stacked promoter:*BvLz* lines with additional *BvLz* expression vectors. The protein accumulation achieved with the combinatorial promoter stacking expression system was stable in multiple vegetative propagations, demonstrating the feasibility of using sugarcane as a biofactory for producing high-value proteins and bioproducts.

## Introduction

Recombinant proteins are currently being produced in cultured cell-based systems in mammals, microbes (bacteria and yeast), insects and plants, as well as in transgenic animals (reviewed by Demain and Vaishnav)^1^. Transgenic plants constitute an attractive system for expression and production of a variety of proteins and biomolecules due to their efficient eukaryotic protein synthesis, high scalability, relatively low production costs and environmental footprint^2–4^. However, selecting suitable hosts and expression vectors are key considerations since protein accumulation is determined by expression levels.

Important factors to consider when selecting a plant-based production platform include biomass yield per hectare, recombinant protein yield per unit biomass, ease of transformation, scalability and safety^5^. Sugarcane (*Saccharum* spp. hybrids), a key feedstock in the expanding bioeconomy as a sugar and bioenergy crop^6^, is an ideal platform for recombinant protein production for several reasons: (1) It is a relatively fast growing tropical grass with the highly efficient C_4_ photosynthetic pathway, conferring high biomass production capacity with yields of up to 41.3 tons of biomass (harvested dry mass) per hectare per annum^7,8^; (2) it is highly efficient in utilizing radiation, water and nutrients to produce a large biomass and hence a higher recombinant protein yield; (3) it is readily amenable to genetic engineering, with established transformation and tissue regeneration techniques^9,10^; and (4) it has a low risk of out-crossing recombinant genes due to its primarily vegetative means of propagation; natural reproductive propagation in many temperate and subtropical regions is rare due to its photoperiod sensitivity.

Sugarcane was used as biofactory for the production of new biomolecules such as bioplastics^11–15^, alternative sugars (sorbitol and isomaltulose)^16–18^, and recombinant proteins including the human cytokine granulocyte macrophage colony stimulating factor GM-CSF^19^, canecystatins (cysteine protease inhibitors) CaneCP-1, CaneCP-2 and CaneCP-3^20–22^, and the cellulolytic enzymes, endoglucanase and cellobiohydrolases I and II^23,24^. Accumulation levels of these recombinant proteins ranged from 0.02 to 2.0% of total soluble protein (TSP) in leaves. However, very few attempts have so far been made to express recombinant proteins in sugarcane culms (reporter proteins)^25^, which constitute the largest fraction of harvestable biomass and would be an ideal platform for production of bulk proteins.

Bovine lysozyme (BvLz) is more important industrially than other lysozymes because of its potent broad-spectrum antimicrobial activity^26,27^, especially against Gram-negative bacteria and fungi at concentrations as low as 25 ppm, its sixfold higher chitinase activity than that of chicken lysozyme^28^, and its thermal stability and resistance to proteolysis^29^. BvLz, unlike other enzymes, possesses biochemical properties that make it suitable for protein extraction and purification, such as stability over a broad pH range, thermal stability, resistance to proteolysis and convenient quantification assays^30,31^.

In this study, we demonstrate the feasibility of developing sugarcane as an expression platform for production and purification of recombinant proteins at high levels, i.e. up to 11.5% of TSP (82.5 mg protein/kg culm mass). Multiple promoters (constitutive or culm-regulated) on separate expression vectors were stacked by combinatorial plant transformation approach to boost production levels of recombinant *bovine lysozyme* (*BvLz*), which was codon-optimized for expression in monocots. A double terminator or 3′ untranslated region (UTR) was incorporated for improved transcript stability. Enzymatic activity and enzyme-linked immunosorbent assays (ELISA) of *BvLz* transgenic sugarcane culm protein extracts and clarified juice confirmed the presence of an intact and fully active BvLz enzyme, which accumulated in multiple vegetative generations at levels as high as 10.0 mg/kg (1.4% of TSP) in lines co-expressing *BvLz* from stacks of three or four different promoters on separate vectors, compared to 0.56 mg/kg (0.07% of TSP) in lines expressing *BvLz* from a single promoter vector. We further observed BvLz accumulation up to 82.5 mg/kg (11.5% of TSP) through event stacking by re-transforming the stacked promoter:*BvLz* transgenic lines with additional *BvLz* expression vectors.

## Results and discussion

### The combinatorial promoter and event stacking result in increased recombinant protein production in transgenic sugarcane culms

A salient feature of combinatorial transformation, a special case of co-transformation^32^, is that there is no theoretical limit to the number of expression vectors that can be co-transformed. To enable high-levels of recombinant protein production in sugarcane culms, we developed a combinatorial promoter and event stacking system and demonstrated its application in producing a high-value bovine lysozyme (BvLz) protein. This was facilitated by the availability of a set of constitutive and culm-regulated promoters previously isolated from sugarcane, in addition to the common maize *ubiquitin 1* promoter (pUbi)^33^. These include the culm-regulated promoters for *Sugarcane bacilliform virus* (pSCBV21)^34^ and sugarcane *dirigent16* (pSHDIR16) gene^35^, and the constitutive promoters for sugarcane *proline-rich protein* (pSHPRP)^36^ and *elongation factor 1α* (pSHEF1α)^36^ genes. Furthermore, conditions for small-scale and large-scale extraction and clarification of recombinant BvLz from sugarcane culm extracts and juice were optimized at our Pilot Plant and BioSeparation Facilities^30,37^.

**Figure 1:**
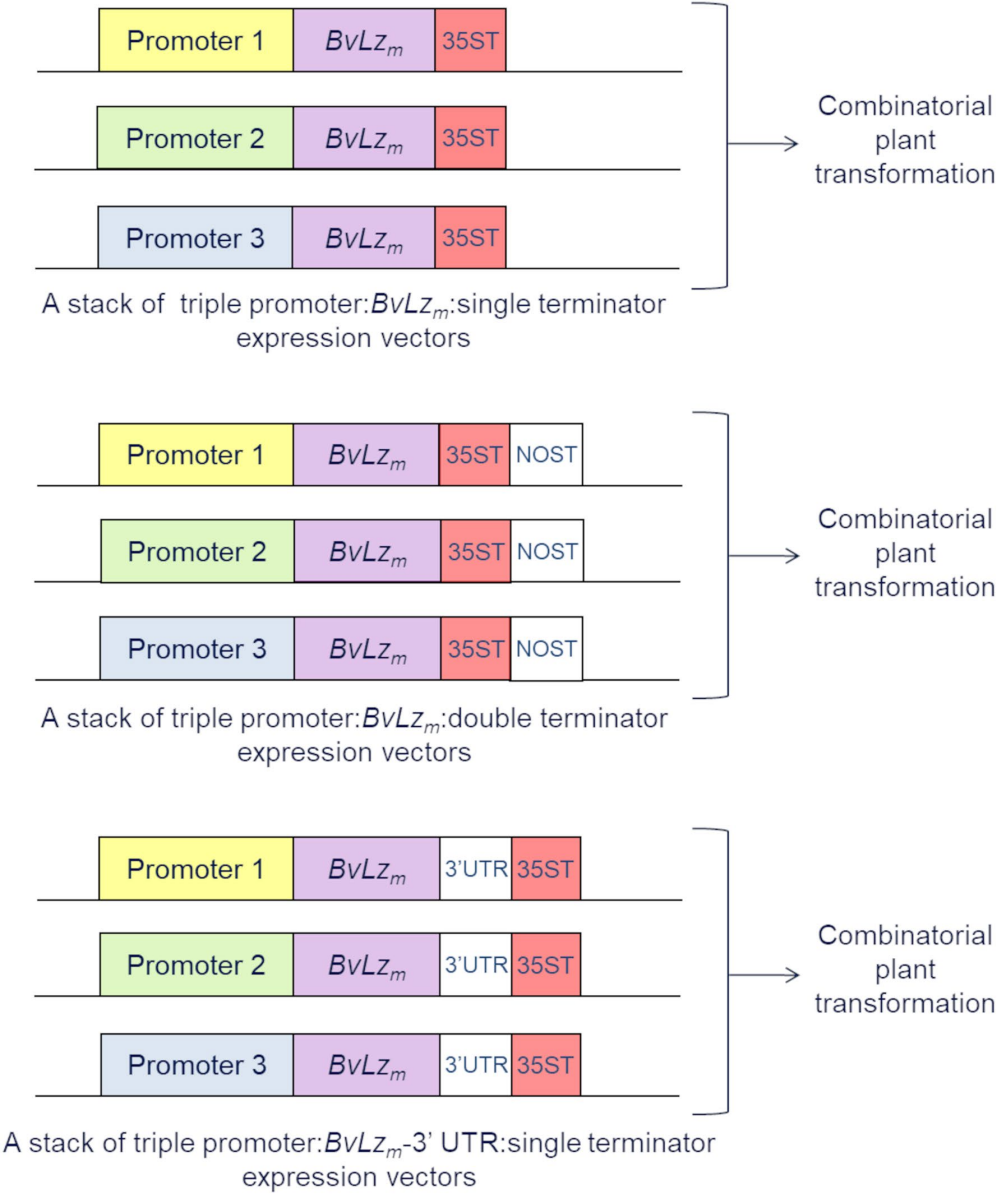
Design of a representative stacked multiple promoter:recombinant gene expression system developed for sugarcane. Promoter 1, 2 and 3 combinations can be any combination of the constitutive promoters maize, *ubiquitin 1*, sugarcane *proline rich protein* and sugarcane *elongation factor 1α* or the culm-regulated promoters from sugarcane *dirigent16* and *Sugarcane bacilliform virus*. Vector assembly and cloning sites are indicated under “Materials and methods” section. *BvLz*_*m*_, maize codon-optimized *bovine lysozyme* gene; 35ST, terminator derived from *Cauliflower mosaic virus* 35S RNA; NOST, *Agrobacterium tumefaciens* nopaline synthase terminator; 3′UTR, 3′ untranslated region of *Sorghum mosaic virus*.

The essential design of the resulting new combinatorial promoter stacking system is illustrated in Fig. 1. The system consisted of co-expressing the codon-optimized *BvLz*_*m*_, from a stack of multiple promoters on separate expression vectors in sugarcane by combinatorial transformation. A double terminator, composed of the *Cauliflower mosaic virus* (CaMV) 35S terminator (35ST) and the *Agrobacterium tumefaciens* nopaline synthase terminator (NOST), or the 3′UTR of *Sorghum mosaic virus* (SrMV), was fused to the coding region of *BvLz*_*m*_ to enhance transcript stability^38,39^ (Fig. 1).

**Figure 2:**
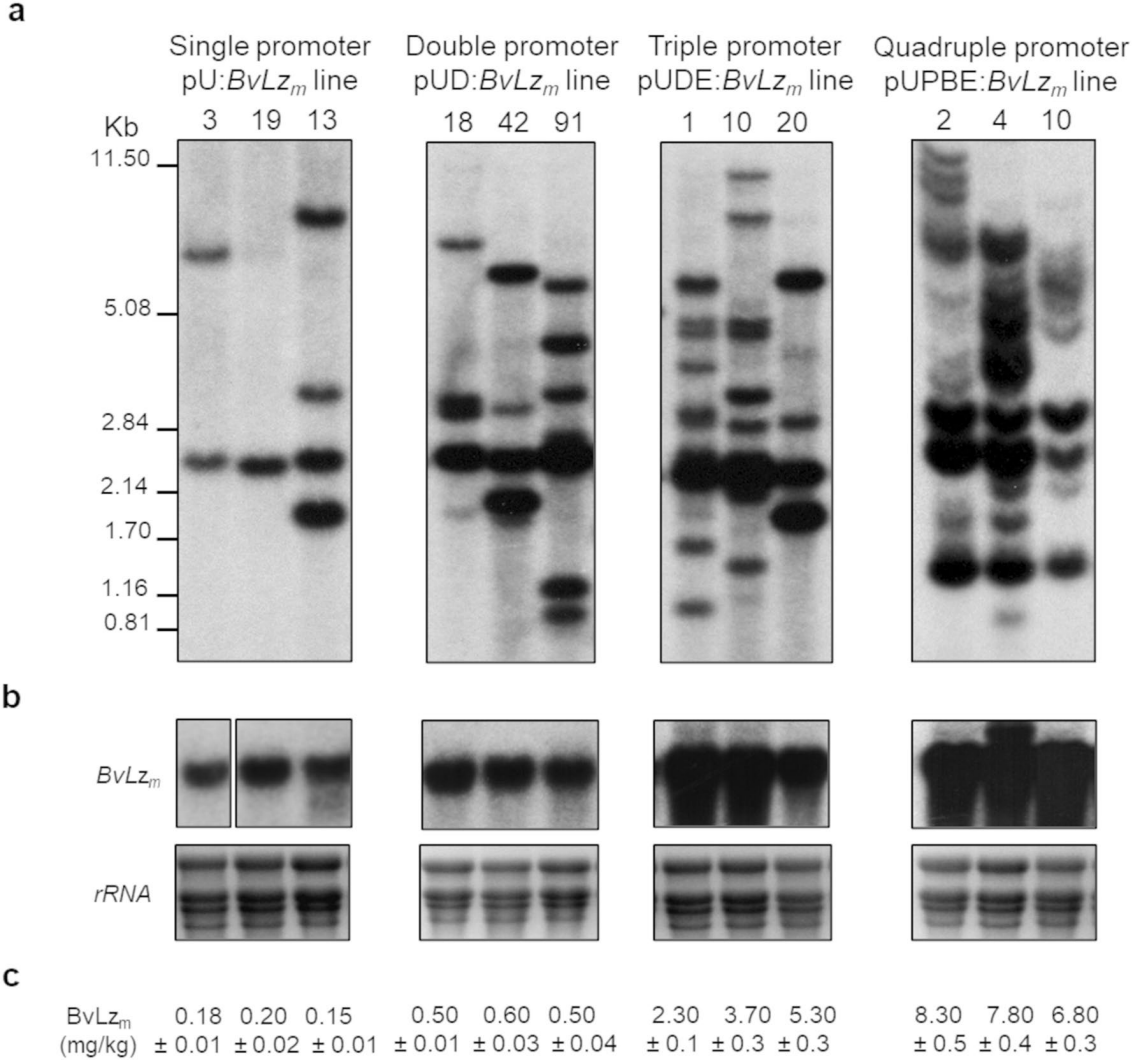
Stable integration, expression and yield of the *bovine lysozyme* (*BvLz*_*m*_) recombinant gene in sugarcane *BvLz*_*m*_ transgenic lines as determined by Southern (**a**) and northern (**b**) blot analyses and enzyme-linked immunosorbent assay (ELISA) (**c**), respectively. Representative lines with single or multiple promoter:*BvLz*_*m*_-terminator cassettes are shown. *BvLz*_*m*_, maize codon-optimized *BvLz*; pU:*BvLz*_*m*_, *BvLz*_*m*_ driven by the maize *ubiquitin 1* promoter (pU); pUD:*BvLz*_*m*_, *BvLz*_*m*_ expressed from two promoters, pU and sugarcane *dirigent16 *(pD); pUDE:*BvLz*_*m*_, *BvLz*_*m*_ expressed from three promoters, pU, pD and sugarcane *elongation factor 1α* (pE); and pUPBE:*BvLz*_*m*_, *BvLz*_*m*_ expressed from four promoters, pU, sugarcane *proline-rich protein* (pP), *Sugarcane bacilliform virus* (pB) and pE. DNA and RNA gel blots were hybridized to a probe corresponding to the coding region of *BvLz*_*m*_. The full-length uncropped DNA and RNA gel blot autoradiograms are displayed in Supplementary Figures S2 and S3, respectively. The BvLz_m_ yield is indicated as determined by ELISA in juice extract of culms (1.0 kg of culm).

To test the stacking promoter gene expression system, embryogenic calli (2 month-old) and leaf roll discs (12 day-old), prepared from several commercial sugarcane varieties were co-transformed biolistically with the multiple promoter:*BvLz*_*m*_ expression vectors, using the *bar* gene (phosphinothricin acetyl transferase) as a selectable marker. Several independent transgenic *BvLz*_*m*_ lines, identified by Southern blot analysis (Fig. 2a; Supplementary Fig. S2), were generated from the combinatorial transformation of sugarcane with single, double, triple or quadruple promoter:*BvLz*_*m*_ expression vectors (Table 1). These represent: (1) 43 lines (114 plants) expressing *BvLz*_*m*_ from a single promoter, (2) 10 lines (52 plants) expressing *BvLz*_*m*_ from a double promoter stack, (3) 24 lines (318 plants) expressing *BvLz*_*m*_ from a triple promoter stack, and (4) 23 lines (76 plants) expressing *BvLz*_*m*_ from a quadruple promoter stack (Table 1).

**Table 1:** Recombinant bovine lysozyme yield of transgenic sugarcane culm.

*Bovine lysozyme* (*BvLz*_*m*_) expressing line and percentage of plants	BvLz_m_ yield as determined by ELISA	
	BvLz_m_ (mg/kg culm mass)	TSP (%)
Promoter stacking		
*1. Single promoter:BvLz*_*m*_* lines*		
pU:*BvLz*_*m*_:single terminator (35ST) lines (43 lines; 114 plants)	0.08–0.4 (range)	0.01–0.06 (range)
6.70%	0.08–0.1	0.01–0.015
40.00%	0.12–0.18	0.02–0.027
53.30%	0.2–0.4	0.03–0.06
*2. Double promoter:BvLz*_*m*_* lines*		
pUD:*BvLz*_*m*_:single terminator (35ST) lines (10 lines; 52 plants)	0.5–0.7 (range)	0.07–0.1 (range)
71.00%	0.5–0.58	0.07–0.077
29.00%	0.6–0.7	0.08–0.1
*3. Triple promoter:BvLz*_*m*_* lines*		
pUPE:*BvLz*_*m*_:3′UTR–single terminator (35ST) (10 lines; 32 plants)	1.0–4.7 (range)	0.1–0.7 (range)
66.70%	1.0–2.0	0.1–0.3
20.00%	2.2–3.2	0.33–0.45
13.30%	3.5–4.7	0.5–0.7
pUDE:*BvLz*_*m*_:3′UTR–single terminator (35ST) (14 lines; 286 plants)	1.5–6.0 (range)	0.2–0.8 (range)
27.00%	1.5–2.0	0.2–0.3
62.00%	2.2–3.2	0.33–0.45
4.50%	3.5–4.7	0.5–0.7
6.50%	5.0–6.0	0.7–0.8
*4. Quadruple promoter:BvLz*_*m*_* lines*		
pUPBE:*BvLz*_*m*_:3′UTR-single terminator (35ST) (12 lines; 36 plants)	2.0–6.3 (range)	0.3–0.9 (range)
33.30%	2.0–3.5	0.3–0.5
22.20%	4.0–5.5	0.6–0.77
44.50%	6.0–6.3	0.8–0.9
pUPBE:*BvLz*_*m*_:double terminator (35STNOST) (11 lines; 40 plants)	2.4–10.0 (range)	0.3–1.4 (range)
24.20%	2.4–3.5	0.3–0.5
51.70%	4.0–5.5	0.6–0.77
24.10%	6.0–10.0	0.8–1.4
**Event stacking**		
*Five promoter:BvLz*_*m*_* lines*		
pUDE:*BvLz*_*m*_ line + pP:*BvLz*_*m*_ + pB:*BvLz*_*m*_ (44 lines; 110 plants)	11.0–82.5 (range)	1.5–11.5 (range)
12.10%	11.0–12.4	1.5–1.7
24.20%	15.9–21.1	2.2–2.9
33.30%	26.2–32.3	3.6–4.5
18.30%	59.9–82.5	8.3–11.5

The percentage (%) of lines with the corresponding BvLz_*m*_ yield are indicated for each single or stacked promoter: *BvLz*_*m*_ construct.

*BvLz*_*m*_, maize codon-optimized *bovine lysozyme* gene; U, maize *ubiquitin 1* promoter; D, sugarcane *dirigent16* promoter; P, sugarcane *proline rich protein* promoter; E, sugarcane *elongation factor 1α* promoter; B, *Sugarcane bacilliform virus* promoter; 3′UTR, 3′ untranslated region of *Sorghum mosaic virus*; 35ST, *Cauliflower mosaic virus* 35S terminator; NOST, *Agrobacterium tumefaciens* nopaline synthase terminator; ELISA, enzyme-linked immunosorbent assay; TSP, total soluble protein.

**Figure 3:**
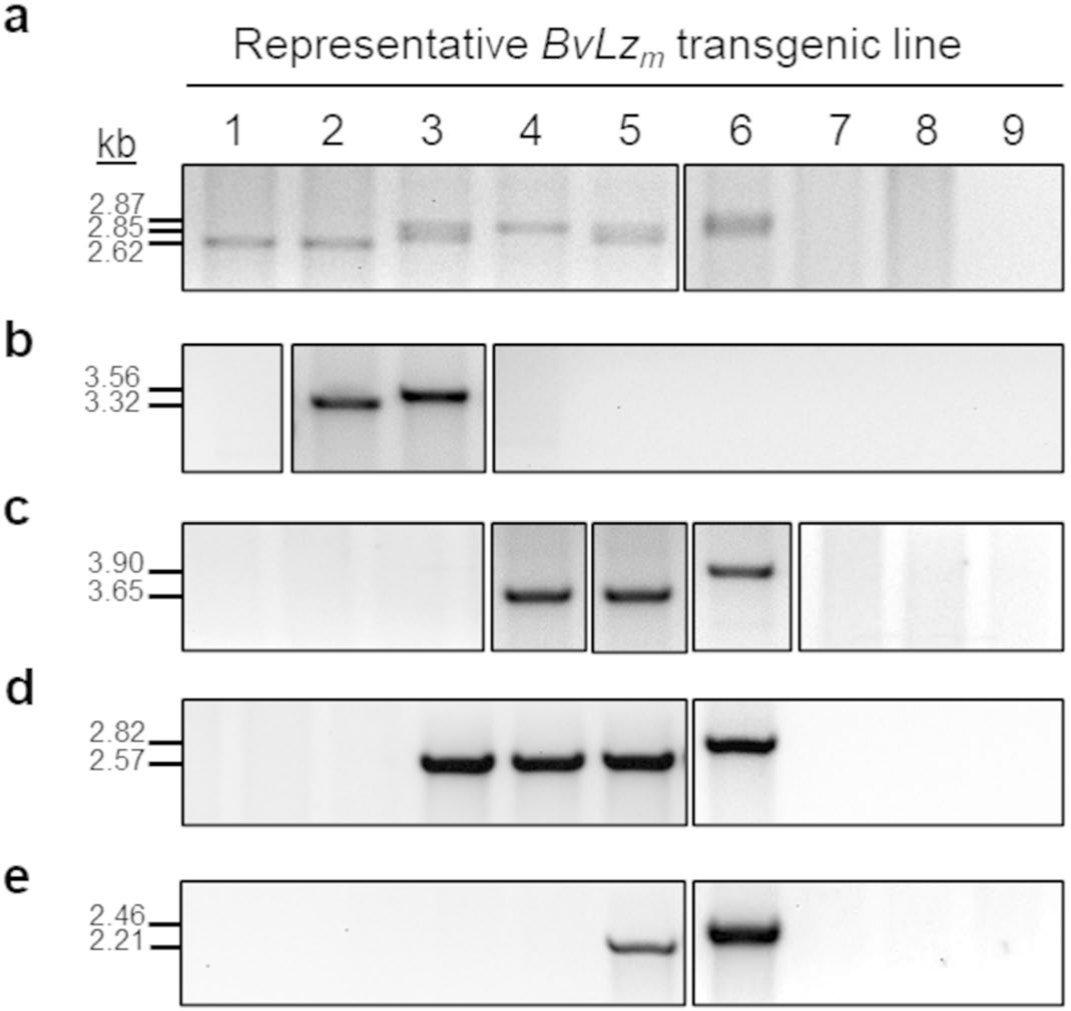
Presence and size of multiple promoter:*bovine lysozyme* (*BvLz*_*m*_)-terminator cassettes in the same *BvLz*_*m*_ transgenic line as determined by PCR analysis. Representative lines with single or multiple promoter:*BvLz*_*m*_-terminator cassettes are shown. (1) pU:*BvLz*_*m*_*-*35ST line; (2) pUD:*BvLz*_*m*_*-*35ST line; (3) pUDE:*BvLz*_*m*_*-*3′UTR-35ST line; (4) pUPE:*BvLz*_*m*_*-*3′UTR-35ST line; (5) pUPBE:*BvLz*_*m*_*-*3′UTR-35ST line; (6) pUPBE:*BvLz*_*m*_*-*35STNOST line; (7) vector-transformed line; (8) non-transformed (NT; tissue culture-derived) plant; and (9) no DNA template (negative control for PCR). (**a**) Detection of pUbi, *BvLz*_*m*_, 3′UTR, 35ST and NOST using the primer sets pUbi-F/35ST-R (2.62 kilobase pairs [kb] or 2.85 kb fragment) and pUbi-F/NOST-R (2.87 kb fragment). (**b**) Detection of pSHDIR16, *BvLz*_*m*_, 3′UTR, 35ST and NOST using the primer sets pSHDIR16-F/35ST-R (3.32 kb fragment) and pSHDIR16-F/NOST-R (3.56 kb fragment). (**c**) Detection of pSHPRP, *BvLz*_*m*_, 3′UTR, 35ST and NOST using the primer sets pSHPRP-F/35ST-R (3.65 kb fragment) and pSHPRP-F/NOST-R (3.90 kb fragment). (**d**) Detection of pSHEF1α, *BvLz*_*m*_, 3′UTR, 35ST and NOST using the primer sets pSHEF1α-F/35ST-R (2.57 kb fragment) and pSHEF1α-F/NOST-R (2.82 kb fragment). (**e**) Detection of pSCBV21, *BvLz*_*m*_, 3′UTR, 35ST and NOST using the primer sets pSCBV21-F/35ST-R (2.21 kb fragment) and pSCBV21-F/NOST-R (2.46 kb fragment). *BvLz*_*m*_, maize codon-optimized *bovine lysozyme* gene; U, Ubi promoter; D, SHDIR16 promoter; P, SHPRP promoter; E, SHEF1α promoter; B, SCBV21 promoter; 3′UTR, 3′ untranslated region of *Sorghum mosaic virus*. 35ST, *Cauliflower mosaic virus* 35S terminator; NOST, *Agrobacterium tumefaciens* nopaline synthase terminator. Full-length uncropped gels of the PCR products are displayed in Supplementary Figures S4, S5 and S6.

The integration and size of each respective *BvLz*_*m*_ expression vector (promoter, *BvLz*_*m*_, terminator and/or 3′UTR) in the single and stacked multiple promoter:*BvLz*_*m*_ lines were confirmed by Southern blot hybridization with a full-length *BvLz*_*m*_ probe (Fig. 2a; Supplementary Fig. S2) and by PCR using primers encompassing each of the different promoter:*BvLz*_*m*_-terminator cassettes (Fig. 3; Supplementary Fig. S4, S5 and S6). All lines were analyzed for their *BvLz*_*m*_ transcript levels by northern blot hybridization (Fig. 2b; Supplementary Fig. S3) as well as for their BvLz_m_ accumulation by ELISA (Table 1; Fig. 2c for ELISA). For representative lines, yield was also determined by an enzyme activity assay and the results highly correlated with the ELISA data (R = 0.81–0.98; Supplementary Table S1). Furthermore, in general, a clear positive trend was observed between the *BvLz*_*m*_ copy number, the combinatorial promoter-*BvLz*_*m*_ cassettes transformed and the BvLz_m_ levels (Table 2; Fig. 2; Supplementary Fig. S2). For instance, quadruple and triple promoter:*BvLz*_*m*_ lines displayed a higher *BvLz*_*m*_ copy number and yield than double and single promoter:*BvLz*_*m*_ lines, as expected from co-transformation (Table 2; Fig. 2; Supplementary Fig. S2). Similarly, the double promoter pUD:*BvLz*_*m*_ lines had a higher *BvLz*_*m*_ copy number and accumulation than single promoter pU:*BvLz*_*m*_ lines (Table 2; Fig. 2; Supplementary Fig. S2).

**Table 2:** Average gene copy number for representative *bovine lysozyme* expressing lines as determined by quantitative PCR.

*Bovine lysozyme *(*BvLz*_*m*_) expressing line	BvLz_m_ yield (mg/kg culm mass)	Average *BvLz*_*m*_ copy number				
		pU-*BvLz*_*m*_ junction	pD-*BvLz*_*m*_ junction	pP-*BvLz*_*m*_ junction	pE-*BvLz*_*m*_ junction	pB-*BvLz*_*m*_ junction
pU:*BvLz*_*m*_-single terminator (35ST)						
3	0.18 ± 0.01	4.0 ± 0.2	0.0 ± 0.0	0.0 ± 0.0	0.0 ± 0.0	0.0 ± 0.0
13	0.20 ± 0.02	1.0 ± 0.1	0.0 ± 0.0	0.0 ± 0.0	0.0 ± 0.0	0.0 ± 0.0
19	0.15 ± 0.01	2.0 ± 0.1	0.0 ± 0.0	0.0 ± 0.0	0.0 ± 0.0	0.0 ± 0.0
pUD:*BvLz*_*m*_-single terminator (35ST)						
18	0.50 ± 0.01	6.0 ± 0.3	4.0 ± 0.2	0.0 ± 0.0	0.0 ± 0.0	0.0 ± 0.0
42	0.60 ± 0.03	7.0 ± 0.3	5.0 ± 0.2	0.0 ± 0.0	0.0 ± 0.0	0.0 ± 0.0
91	0.50 ± 0.04	6.0 ± 0.2	7.0 ± 0.2	0.0 ± 0.0	0.0 ± 0.0	0.0 ± 0.0
pUPE:*BvLz*_*m*_-3′UTR-35ST						
11	1.4 ± 0.1	6.0 ± 0.3	0.0 ± 0.0	18.0 ± 4.1	6.0 ± 0.2	0.0 ± 0.0
22	2.0 ± 0.2	6.0 ± 0.2	0.0 ± 0.0	15.0 ± 0.5	3.0 ± 0.4	0.0 ± 0.0
24	3.5 ± 0.3	5.0 ± 0.1	0.0 ± 0.0	11.0 ± 0.6	4.0 ± 0.4	0.0 ± 0.0
pUDE:*BvLz*_*m*_-3′UTR-35ST						
1	2.3 ± 0.1	8.0 ± 0.8	13.0 ± 1.5	0.0 ± 0.0	6.0 ± 0.5	0.0 ± 0.0
10	3.7 ± 0.3	6.0 ± 0.4	10.0 ± 0.9	0.0 ± 0.0	5.0 ± 0.1	0.0 ± 0.0
20	5.3 ± 0.3	10.0 ± 1.0	8.0 ± 0.6	0.0 ± 0.0	3.0 ± 0.3	0.0 ± 0.0
pUPBE:*BvLz*_*m*_-3′UTR-35ST						
1	6.3 ± 0.4	10.0 ± 0.9	0.0 ± 0.0	17.0 ± 1.6	6.0 ± 0.3	15.0 ± 1.2
4	6.0 ± 0.3	5.0 ± 0.3	0.0 ± 0.0	20.0 ± 2.6	4.0 ± 0.1	6.0 ± 0.4
15	6.0 ± 0.2	6.0 ± 0.4	0.0 ± 0.0	12.0 ± 1.4	8.0 ± 0.4	11.0 ± 0.7
pUPBE:*BvLz*_*m*_-35STNOST						
1	6.7 ± 0.3	6.0 ± 0.3	0.0 ± 0.0	30.0 ± 1.4	7.0 ± 0.8	18.0 ± 1.6
2	10.0 ± 0.7	3.0 ± 0.2	0.0 ± 0.0	11.0 ± 0.4	3.0 ± 0.2	7.0 ± 0.7
4	8.3 ± 0.4	11.0 ± 0.9	0.0 ± 0.0	25.0 ± 1.6	5.0 ± 0.3	25.0 ± 2.6
pUDE:*BvLz*_*m*_ + pP:*BvLz*_*m*_ + pB:*BvLz*_*m*_						
1	11.8 ± 1.4	10.0 ± 1.3	23.0 ± 3.0	15.0 ± 1.6	8.0 ± 0.9	10.0 ± 1.0
5	14.6 ± 2.3	8.0 ± 1.2	13.0 ± 1.4	25.0 ± 2.7	10.0 ± 1.5	22.0 ± 2.4
12	28.6 ± 3.4	14.0 ± 1.5	17.0 ± 1.9	21 ± 2.3	12.0 ± 1.9	31.0 ± 4.1

The gene copy number was estimated based on gene copy number indices generated using the reference gene prolyl 4-hydroxylase.

*BvLz*_*m*_, maize codon-optimized *BvLz*; pU, maize *ubiquitin 1* promoter; pUD:*BvLz*_*m*_, *BvLz*_*m*_ expressed from two promoters, maize *ubiquitin 1* and sugarcane *dirigent16* (pD); pUPE:*BvLz*_*m*_, *BvLz*_*m*_ expressed from three promoters, pU, sugarcane *proline-rich protein* (pP) and sugarcane *elongation factor 1α* (pE); pUDE:*BvLz*_*m*_, *BvLz*_*m*_ expressed from three promoters, pU, pD and pE; pUPBE:*BvLz*_*m*_, *BvLz*_*m*_ expressed from four promoters, pU, pP, pE and *Sugarcane bacilliform virus*; 3′UTR, 3′ untranslated region of *Sorghum mosaic virus*; 35ST, *Cauliflower mosaic virus* 35S terminator; NOST, *Agrobacterium tumefaciens* nopaline synthase terminator. The BvLz_m_ yield is indicated as determined by enzyme-linked immunosorbent assay in juice extract of culms (one kg of culm).

The BvLz_m_ yield from single promoter pUbi:*BvLz*_*m*_ (pU:*BvLz*_*m*_) lines varied from low (0.08–0.1 mg/kg; 6.7% of plants) to moderate (0.12–0.18 mg/kg; 40.0% of plants) and high (0.2–0.4 mg/kg; 53.3% of plants) (Tables 1, 2). The BvLz_m_ yield range was 0.08–0.4 mg/kg (0.01–0.06% of TSP), averaging 0.3 mg/kg (0.04% of TSP) ± 0.02 for the high expressers (Table 1). Other single promoter:*BvLz*_*m*_ lines harboring pSHDIR16, pSCBV21, pSHPRP or pSHEF1α showed similar trends, with a highest BvLz_m_ yield of 0.56 mg/kg (0.08% of TSP) (Supplementary Table S2).

Stacked double promoter pUbi-SHDIR16:*BvLz*_*m*_ (pUD:*BvLz*_*m*_) lines displayed 1.8–6.3 fold higher BvLz_m_ yield than single promoter pU:*BvLz*_*m*_ lines, with a range of 0.5–0.7 mg/kg (0.07–0.1% of TSP) (Table 1). The BvLz_m_ yield was further enhanced to 2.0–8.6 fold in the stacked triple promoter:*BvLz*_*m*_ lines, with levels ranging from 1.0 to 6.0 mg/kg (0.1–0.8% of TSP) (Table 1). The majority (66.7%) of the stacked triple promoter pUbi-SHPRP-SHEF1α:*BvLz*_*m*_ (pUPE:*BvLz*_*m*_) lines had a BvLz_m_ yield of 1.0–2.0 mg/kg (0.1–0.3% of TSP) with 20.0% at 2.2–3.2 mg/kg (0.33–0.45% of TSP) and 13.3% at 3.5–4.7 mg/kg (0.5–0.7% of TSP) (Table 1). Replacing the constitutive SHPRP promoter with the culm-regulated SHDIR16 promoter in the stacked triple promoter pUbi-SHDIR16-SHEF1α:*BvLz*_*m*_ (pUDE:*BvLz*_*m*_) lines boosted the BvLz_m_ yield to 6.0 mg/kg (0.8% of TSP). Most of pUDE:*BvLz*_*m*_ lines (62.0%) had a BvLz_m_ yield of 2.2–3.2 mg/kg (0.33–0.45%of TSP), with 27.0% at 1.5–2.0 mg/kg (0.2–0.3% of TSP), 4.5% at 3.5–4.7 mg/kg (0.5–0.7% of TSP) and 6.5% at 5.0–6.0 mg/kg (0.7–0.8% of TSP) (Table 1).

Next, we checked if stacking another promoter to produce quadruple promoter:*BvLz*_*m*_ lines would be helpful. The BvLz_m_ yield increased modestly in the stacked quadruple promoter pUbi-SHPRP-SCBV21-SHEF1α:*BvLz*_*m*_ (pUPBE:*BvLz*_*m*_) lines by 1.7–2.4 fold, compared to the stacked triple promoter:*BvLz*_*m*_ lines. The highest enhancement was achieved when using a double terminator cassette, i.e. 10.0 mg/kg (1.4% of TSP) in pUPBE:*BvLz*_*m*_:35STNOST lines (Table 1), and the 3′UTR of SrMV with the single 35S terminator, i.e. 6.3 mg/kg (0.9% of TSP) in pUPBE:*BvLz*_*m*_:3′UTR35ST lines (Table 1). In fact, 24.1% of pUPBE:*BvLz*_*m*_:35STNOST plants had a BvLz_m_ yield of 6.0–10 mg/kg (0.8–1.4% of TSP), and 44.5% of pUPBE:*BvLz*_*m*_:3′UTR35ST plants showed a BvLz_m_ yield of 6.0–6.3 mg/kg (0.8–0.9% of TSP) (Table 1). Lastly, we evaluated if event stacking can enhance the yields of the stacked quadruple promoter lines. Event stacking, also referred to as super transformation, is a good alternative to hybridization/crossing, which is time-consuming and not a viable option in vegetatively-propagated crops like sugarcane. Stacked five promoter pUbi-SHDIR16-SHEF1α-SHPRP-SCBV21:*BvLz*_*m*_ (pUDEPB:*BvLz*_*m*_) lines were generated through event stacking, by re-transforming bialaphos-resistant triple promoter pUDE:*BvLz*_*m*_ lines with two promoter:*BvLz*_*m*_ expression vectors, pP:*BvLz*_*m*_ and pB:*BvLz*_*m*_ (Table 1) using the *neomycin phosphotransferase II* as a selectable marker. The resulting pUDEPB:*BvLz*_*m*_ lines showed increased BvLz_m_ accumulation, i.e. up to 82.5 mg/kg culm mass (11.5% of TSP) (Table 1). The majority (33.3%) of these lines exhibited BvLz_m_ levels of 26.2–32.3 mg/kg (3.6–4.5% of TSP), while 18.3% accumulated the highest BvLz_m_ levels, i.e. 59.9–82.5 mg/kg (8.3–11.5% of TSP). The remaining 24.2% and 12.1% of the lines showed BvLz_m_ levels of 15.9–21.1 mg/kg (2.2–2.9% of TSP) and 11.0–12.4 mg/kg (1.5–1.7% of TSP), respectively (Table 1). Notably, BvLz_m_ accumulation was highly enhanced in the new stacked five promoter pUDEPB:*BvLz*_*m*_ lines by 7.3–13.8-fold, compared to the receiving stacked triple promoter pUDE:*BvLz*_*m*_ lines. Together, these experiments demonstrate that high levels of recombinant BvLz_m_ (up to 11.5% of TSP or 82.5 mg/kg) can be successfully produced in sugarcane culms using the combinatorial promoter and event stacking strategies. Previous studies utilized multiple plant species, tissue types, and expression systems for recombinant protein production^40,41^. Majority of them used transient *Agrobacterium*- and viral vector-based approaches in *Nicotiana benthamiana* or *N. tabacum*^42–46^. While the transient systems are viable approaches, they are technically feasible only in few plant species that are amenable for infiltration and/or are hosts for the viruses used as viral vectors. In this context, transgenic plant systems are more suited for wider adoption since broad range of plant species can be transformed using latest biotechnology tools. When comparing our results of protein expression in sugarcane culms with other transgenic plant expression systems, caution was exercised particularly when comparing recovered protein yields per starting tissue weight (e.g., mg/kg). This is because not all plant tissues have similar compositions, nor the protein extractions are equally efficient among tissue types, owing to biological and biochemical differences^41^. For instance, sugarcane culms primarily constitute juice (sugars) and lignocellulosic fiber (bagasse). An equal amount of *N. benthamiana* leaves on a fresh weight basis will have less fiber, and proteins may be easier to extract from leaf tissues. We also note that biochemical properties of target proteins such as size, solubility, amino-acid composition, structural features, and protein stability may also ultimately influence the final yield. With these caveats in mind, we compared our results with other reported studies of transgenic plant systems using the % TSP unit of recovered proteins. Several studies have reported recombinant protein yields of ~ 0.002 to 0.05% of TSP in transgenic carrots^47,48^, ~ 0.23–2.5% of TSP in transgenic tobacco and potato^49^, ~ 8% TSP in transgenic tomato^50^, and ~ 11.9% in transgenic rice^51^. These comparisons suggest that higher protein yields can be achieved using the sugarcane transgenic system (up to 11.5% of TSP), which are comparable to other transgenic systems, if not greater.

In addition to the use of constitutive or tissue-specific promoters, inducible promoters can be used for expressing recombinant proteins in plants^52–54^. Several inducible promoters can be used for generating transgenic plants such as dexamethasone-, ethylene-, heat shock- and estradiol-inducible promoters^52^. Indeed, we have previously shown that the sugarcane DIRIGENT (SHDIR16) promoter is responsive to plant hormones such as salicylic acid or jasmonic acid^35^. This is promising and suggests that inducible promoters such as SHDIR16, and other well-characterized plant inducible-promoters^52^ can be further used in lieu or in combination with the constitutive/tissue-specific promoters that we have described, in order to robustly control and/or fine-tune the recombinant protein expression.

### Increased protein levels were associated with the number of combinatorial stacked promoters and not with the copy number alone

Our results show that using multiple different promoters to drive expression of recombinant *BvLz*_*m*_ on distinct vectors enhanced recombinant protein accumulation. It is possible that the enhanced levels may have occurred due to higher number of inserted *BvLz*_*m*_ copies alone or it could be due to a combination of promoter-driven synergistic transcriptional activity. To test these scenarios, we performed a comparison of the BvLz_m_ transcript and yield among the various promoter stacked lines that had similar number of insertions. This analysis showed that there is a positive correlation in BvLz_m_ transcript and yield with combinatorial promoter:*BvLz*_*m*_ stacks, irrespective of the number *BvLz*_*m*_ inserts (Fig. 2; Supplementary Fig. S2). For instance, for single promoter:*BvLz*_*m*_ line 13, double promoter:*BvLz*_*m*_ line 42, triple promoter:*BvLz*_*m*_ line 20 and quadruple promoter:*BvLz*_*m*_ line 10, with all of them having about 4–5 *BvLz*_*m*_ inserts, there was a clear enhancement in the BvLz_m_ yield (Fig. 2a,c; Supplementary Fig. S2). Conversely, a comparison of single promoter:*BvLz*_*m*_ transgenic lines with one or multiple inserts showed that there was no corresponding increase in BvLz yield with the copy number. For instance, line 19 with one insert (Fig. 2a,c; Supplementary Fig. S2) had a BvLz_m_ yield of 0.2 mg/kg, while line 13 with 4 *BvLz*_*m*_ inserts had a BvLz_m_ yield of 0.15 mg/kg (Fig. 2a, c; Supplementary Fig. S2). Together, these results suggest that the increase in BvLz_m_ yield is primarily attributed to the number of combinatorial stacked multiple promoters and not just with the *BvLz*_*m*_ copy number alone.

### Combinatorial promoter stacking may alleviate transcriptional occlusion and/or recombinant gene silencing

Multiple identical copies of recombinant genes or promoter transcription units (PTUs) delivered through a single construct could trigger transgene silencing^55–57^ or result in promoter occlusion or transcriptional interference, a phenomenon observed in eukaryotic systems, including plants^58–62^. For instance, a strong PTU can sequester most of the transcription factors in its immediate vicinity, limiting transcription from other promoters present in *cis* on the same vector^63^. Alternatively, homology-dependent DNA methylation within the promoter or in the coding region sequences could result in transgene silencing. For instance, in maize, transgenic lines with four copies of a cellulase gene, under control of tandemly arranged PTUs on the same vector, resulted in lowered expression than those lines with fewer copies^64^.

Our results here showed a positive correlation between the number of combinatorial promoter stacks of recombinant *BvLz*_*m*_ and increase in *BvLz*_*m*_ levels, with no apparent transgene silencing. It is likely that using different promoter sequences in separate vectors may overcome the transgene silencing or transcriptional interference. We suggest that each expression vector in the described stacked multiple promoter:*BvLz*_*m*_ system (Fig. 1) does not negatively affect the others, as shown by a positive correlation between the combinatorial promoter:*BvLz*_*m*_ copy number (Table 2) and enhanced steady-state *BvLz*_*m*_ transcript accumulation (Fig. 2b; Supplementary Fig. S3) and BvLz_m_ activity (Table 1; Fig. 2c).

### Elevated recombinant BvLz_m_ accumulation positively enhances transgenic plant growth

Analysis of the deleterious effects of recombinant protein accumulation on plant physiology and growth is crucial in order to assess the economic feasibility of using transgenic plants as biofactories, and this is largely dependent on the target protein function^65^. In our scenario with BvLz_m_, we found no deleterious effects of enhanced *BvLz*_*m*_ expression on sugarcane growth. On the contrary, several growth characteristics of *BvLz*_*m*_ expressing lines were better than those of non-transformed plants, such as enhanced leaf length, culm height, tiller number, culm biomass and Brix (total soluble solids) (Table 3). These differences were statistically significant (*p* < 0.001 and *p* < 0.0001) in the triple and quadruple promoter:*BvLz*_*m*_ expressing lines (Table 3). For instance, mean culm fresh biomass per plant of the quadruple promoter:*BvLz*_*m*_ expressing lines was nearly 2.5 times greater than that of non-transformed plants. The mean soluble solids content in juice from triple and quadruple promoter:*BvLz*_*m*_ expressing lines was approximately 20% higher than that of non-transformed plants. Similar trends were also observed for leaf length, culm height and tiller density. The enhanced agronomic performance of the transgenic lines suggested that BvLz_m_, which is a well-known antimicrobial protein^27^, could have a growth-promoting or perhaps protective role against pathogens present in the natural growth environment.

**Table 3:** Growth and culm quality characteristics of *bovine lysozyme* expressing sugarcane lines.

*Bovine lysozyme *(*BvLz*_*m*_) expressing line	Bvlz_m_ yield (mg/kg culm fresh mass)	Agronomic parameter				
		Leaf length (cm)	Culm height (cm)	Tiller number	Culm biomass (fresh mass, kg/plant)	Brix (%)
Non-transformed (CP72-1210)	0.0 ± 0.0^a^	75.3 ± 1.4^a^	12.5 ± 0.2^a^	4.0 ± 0.2^a^	7.1 ± 0.3^a^	14.6 ± 0.6^a^
**Single promoter maize*ubiquitin 1:BvLz***_***m***_** expressing lines (CP72-1210)**						
33	0.22 ± 0.01^b^*	74.0 ± 0.5^a^	13.5 ± 0.2^a^	10.0 ± 0.4^b^**	9.1 ± 0.6^b^*	17.2 ± 0.6^b^*
67	0.27 ± 0.01^b^*	72.3 ± 1.1^a^	14.5 ± 0.6^a^	4.0 ± 0.3^a^	7.8 ± 0.5^a^	15.7 ± 0.5^b^*
108	0.33 ± 0.01^b^*	76.8 ± 1.4^a^	18.0 ± 0.3^b^**	5.0 ± 0.2^a^	9.6 ± 0.9^b^*	14.9 ± 0.4^a^
114	0.32 ± 0.01^b^*	70.5 ± 2.2^a^	11.3 ± 0.6^a^	6.0 ± 0.2^b^*	8.2 ± 0.6^b^*	16.8 ± 0.4^b^*
116	0.36 ± 0.01^b^*	74.0 ± 3.3^a^	14.5 ± 1.0^a^	8.0 ± 0.9^b^**	7.0 ± 0.3^a^	17.9 ± 0.7^b^*
123	0.26 ± 0.01^b^*	76.5 ± 1.8^a^	17.1 ± 1.3^b^*	4.0 ± 0.1^a^	8.9 ± 0.5^b^*	15.2 ± 0.5^a^
**Triple promoter:*BvLz***_***m***_** expressing lines**						
pUPE:*BvLz*_*m*_ 32 (CP72-1210)	2.7 ± 0.1^b^**	81.9 ± 1.2^a^	16.3 ± 0.4^b^*	17.0 ± 2.9^b^**	13.1 ± 1.2^b^*	18.4 ± 0.3^b^*
pUDE:*BvLz*_*m*_ (TCP98-4454)						
18	5.1 ± 0.3^b^**	92.3 ± 4.1^b^**	17.3 ± 0.2^b^*	7.0 ± 0.6^b^*	10.5 ± 0.9^b^*	16.7 ± 0.3^b^*
19	4.6 ± 0.2^b^**	95.6 ± 2.5^b^**	18.5 ± 0.4^b^**	7.0 ± 0.2^b^*	11.3 ± 1.0^b^*	15.6 ± 0.4^a^
44	2.9 ± 0.1^b^**	74.8 ± 2.6^a^	14.3 ± 1.4^a^	7.0 ± 1.3^b^*	8.6 ± 0.8^b^*	16.0 ± 0.2^b^*
54	6.0 ± 0.3^b^**	87.2 ± 4.0^b^*	16.6 ± 1.1^b^*	4.0 ± 0.5^a^	10.7 ± 1.1^b^*	17.9 ± 0.6^b^*
**Quadruple promoter:*BvLz***_***m***_** expressing lines**						
pUPBE:*BvLz*_*m*_ (TCP98-4454)						
1	6.7 ± 0.3^b^**	115.3 ± 3.1^b^**	19.4 ± 1.2^b^**	21.1 ± 1.3^b^**	16.4 ± 0.7^b^**	19.1 ± 0.6^b^*
2	10.0 ± 0.7^b^**	201.8 ± 6.4^b^**	32.1 ± 2.4^b^**	17.0 ± 1.1^b^**	20.8 ± 1.0^b^**	18.0 ± 0.7^b^*
4	8.3 ± 0.4^b^**	140.2 ± 5.1^b^**	25.3 ± 1.9^b^**	14.0 ± 0.9^b^**	18.6 ± 0.9^b^**	17.5 ± 0.3^b^*

Morphological parameters (leaf height, culm height and tiller number) of 15 representative single promoter:*bovine lysozyme* (*BvLz*_*m*_) and five representative triple promoter:*BvLz*_*m*_ expressing lines were measured every 2 weeks for 4 months after planting. Culm biomass was determined at 11 months (harvest). The BvLz_m_ yield is indicated as determined by enzyme-linked immunosorbent assay in juice extract of culms at the 11-month harvest (one kg of culm). Total soluble solids (Brix) of extracted culm juice from 11-month-old plants was determined using a refractometer (model PR-101α, Atago U.S.A, Inc., Bellevue, WA). Data represent means from four biological replications ± standard errors. Means are compared column-wise. Values that are significantly different from those of non-transformed at *p* < 0.001 and *p* < 0.0001 are denoted by * and **, respectively. *BvLz*_*m*_: maize codon-optimized *BvLz*; pUPE:*BvLz*_*m*_: *BvLz*_*m*_ expressed from three promoters, maize *ubiquitin 1* (pUbi), sugarcane *proline-rich protein* (pSHPRP) and sugarcane *elongation factor 1α* (pSHEF1α); pUDE:*BvLz*_*m*_: *BvLz*_*m*_ expressed from three promoters, pUbi, sugarcane *dirigent16* and pSHEF1α. pUPBE:*BvLz*_*m*_: *BvLz*_*m*_ expressed from four promoters, pUbi, pSHPRP, pSHEF1α and *Sugarcane bacilliform virus* promoter.

### Recombinant protein accumulation in culms increases with plant age

**Figure 4:**
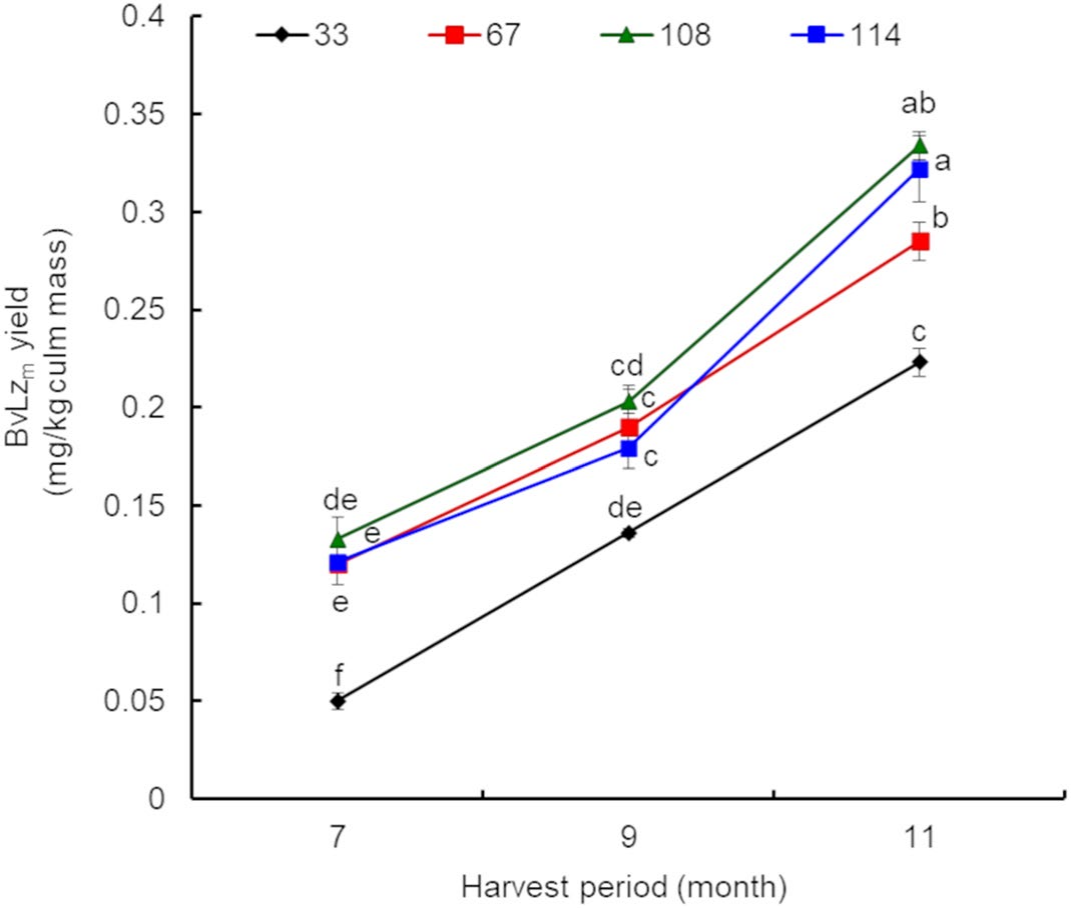
Temporal pattern of recombinant bovine lysozyme (BvLz_m_) accumulation in culms of single promoter:*BvLz*_*m*_ expressing sugarcane lines. BvLz_m_ activity of four representative maize *ubiquitin 1* promoter:*BvLz*_*m*_ lines is shown as determined by enzyme-linked immunosorbent assay in 200.0 ml of juice extract from the 7- and 9-month-harvests, and 650.0–700.0 ml of juice extract from the 11-month-harvest (one kg of culm for all harvests). Values represent four biological samples for each *BvLz*_*m*_ expressing line and are reported with standard errors from three technical replications. *BvLz*_*m*_: maize codon-optimized *BvLz*. Values with different letters are significantly different (*p* < 0.05).

To monitor the temporal stability of BvLz_m_ accumulation in sugarcane culms in a growing season, we analyzed BvLz_m_ levels for 11-months with a selection of several representative single promoter pU:*BvLz*_m_ lines. The BvLz_m_ yields (mg of BvLz_m_/kg of harvested culm) in these lines after 7-, 9- and 11-month-harvest are shown in Fig. 4 (data for four representative lines) and Table 3 (data for six representative lines at the 11 month-harvest). There was a significant (*p* < 0.05) increase in BvLz_m_ yield over time for all the lines tested. BvLz_m_ accumulation was highest at the 11-month harvest, with lines 67, 108 and 114 showing the most significant (*p* < 0.05) increase (Fig. 4). This accumulation pattern coincides with timing of culm ripening, which is characterized by increased sucrose translocation and accumulation in culms. The age-related sucrose accumulation also was associated with the reduction in vegetative development (leaf initiation and expansion) and commences at the mature basal internodes, progressing towards the culm apex, until the entire culm reaches a stable sugar level as it approaches physiological maturity^66^. The age-related pattern of BvLz_m_ accumulation may also be regulated by similar factors whereby photoassimilates and other substrates for BvLz_m_ are diverted from vegetative growth towards metabolite synthesis and accumulation during sugarcane maturation. Regardless of the mechanisms regulating the temporal accumulation of BvLz_m_, our results demonstrate that the recombinant protein levels can be maintained, if not enhanced, during the development phases of sugarcane in a growing season. Similar results were observed for BvLz_m_ accumulation in representative triple promoter:*BvLz*_m_ lines, which showed sustained and stable BvLz_m_ levels over a full growing year, as well as in successive vegetative propagations (Supplementary Table S3). Similar accumulation of recombinant proteins (human therapeutic interleukin-10) with plant maturity was observed in tobacco^67^.

### High level recombinant protein accumulation requires adequate mineral nutrition to sustain the protein and biomass accumulation

**Figure 5:**
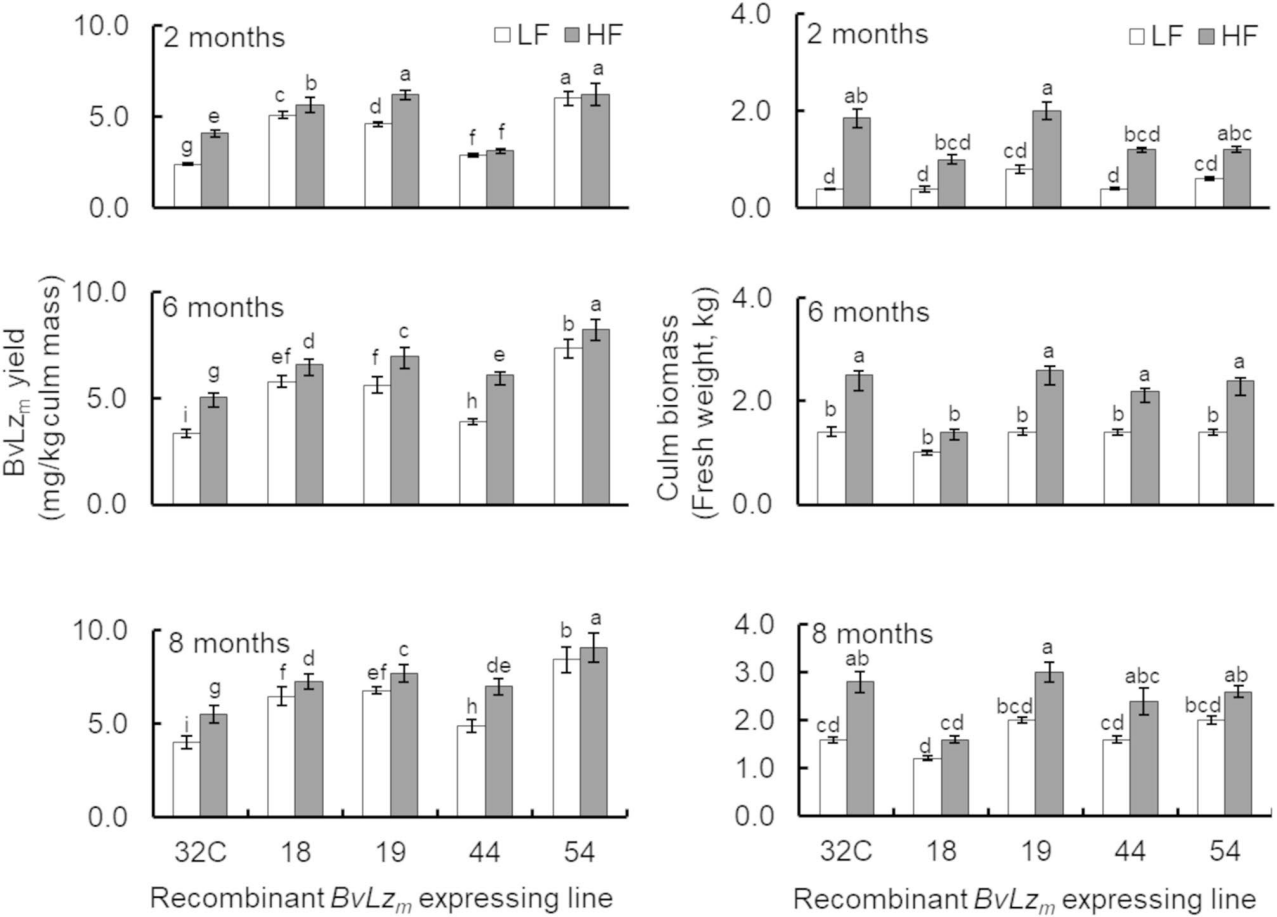
Enhancement of culm biomass and yield of recombinant bovine lysozyme (BvLz_m_) by fertilization in triple promoter:*BvLz*_*m*_ sugarcane lines. BvLz_m_ activity of four representative lines is shown as determined by enzyme-linked immunosorbent assay in juice extract of culms (1.0 kg of culm). Values represented four biological samples and three technical replications at 2, 6 and 8 months following low (LF) or high (HF) fertilization. Values with different letters are significantly different (*p* < 0.05). *BvLz*_*m*_, maize codon-optimized *BvLz*; 32C, pUPE:*BvLz*_*m*_ line; 18, 44 and 54, pUDE:*BvLz*_*m*_ lines; U, maize *ubiquitin 1* promoter; P, sugarcane *proline-rich protein* promoter; E, sugarcane *elongation factor 1α* promoter; and D, sugarcane *dirigent16* promoter.

Adequate water and nutrients supply are important for crop productivity as well as quality considerations, such as protein content and other sensory traits^68^. Because we observed enhanced growth traits such as biomass in the *BvLz*_*m*_ expressing lines, specifically in the triple promoter:*BvLz*_*m*_ lines (Table 3), we next investigated the optimal fertilization regime needed to sustain the additional growth and high levels of BvLz_m_ production. Four representative triple promoter:*BvLz*_*m*_ lines (2-month old) were subjected to two mineral nutrient supply regimes namely, low fertility (LF or 2.4 mg N per plant, twice a week) and a high fertility (HF or 8 mg N per plant), using a balanced commercial fertilizer (Peters Professional 20–20–20; see “Materials and methods” section). BvLz_m_ yield and growth traits were measured at 2-, 6-, and 8-months following fertilization. Supplemental fertilization increased culm biomass and BvLz_m_ yield in the triple promoter:*BvLz*_*m*_ lines over time. The most significant increases (*p* < 0.05) between LF and HF were noted at 2 months for all lines (Fig. 5). For instance, pUPE:*BvLz*_*m*_ line 32C (CP72-1210 variety) and pUDE:*BvLz*_m_ lines 19, 44 and 54 (TCP98-4454 variety) showed 4.6-, 2.5-, 3.0- and 2.0-fold increases in culm biomass and 1.7-, 1.3-, 1.1 and 1.0-fold enhancements in BvLz_m_ yield, respectively.

Leaf macronutrient contents of the triple promoter:*BvLz*_*m*_ plants were also monitored following growth under the two fertilization regimes. Plants grown under high nutrient supply rates had significantly (*p* < 0.0001) higher leaf mineral nutrient contents compared to those grown under low nutrient supply rates (Table 4). Leaves of HF plants had higher levels of N, phosphorus (P), potassium (K) and magnesium (Mg), compared to leaves of LF plants (Table 4). In general, leaf nutrient content of the *BvLz*_*m*_ expressing lines was improved by supplemental fertilization, resulting in a 1.5- to 2.2-fold increase in culm biomass and a subsequent 1.2- to 2.2-fold enhancement in BvLz_m_ yield at 8 month-growth stage (Fig. 5). Taken together, the accumulation of *BvLz*_*m*_ in response to fertilization and the ontogenic *BvLz*_*m*_ accumulation pattern underscore the need for adequate input availability to sustain not only biomass production but also the yield of high-value proteins in crops such as sugarcane.

**Table 4:** Leaf nutrient contents in leaves of triple promoter:*bovine lysozyme* expressing sugarcane lines grown under two fertilization levels.

Line/treatment	Macronutrient content (mg/g tissue dry mass)			
	Nitrogen	Phosphorus	Potassium	Magnesium
pUPE:*BvLz*_*m*_ 32C				
LF	8.8±1.0^a^	1.5 ± 0.2^a^	11.7 ± 0.2^a^	1.4 ± 0.4^a^
HF	15.0 ± 1.0^b^	3.1 ± 0.4^b^	19.5 ± 0.2^b^	3.1 ± 0.3^b^
pUDE:*BvLz*_*m*_ 18				
LF	10.3 ± 0.1^a^	2.2 ± 0.2^a^	17.3 ± 0.3^a^	1.1 ± 0.1^a^
HF	18.7 ± 2.0^b^	4.0 ± 0.2^b^	20.0 ± 1.8^b^	2.1 ± 0.2^b^
pUDE:*BvLz*_*m*_ 19				
LF	12.4 ± 2.0^a^	2.1 ± 0.2^a^	17.3 ± 0.5^a^	1.1 ± 0.1^a^
HF	16.8 ± 1.0^b^	3.3 ± 0.3^b^	19.5 ± 0.7^b^	3.0 ± 0.2^b^
Non-transformed				
LF	9.6 ± 1.5^a^	1.7 ± 0.2^a^	13.0 ± 0.4^a^	1.8 ± 0.2^a^
HF	15.0 ± 2.1^b^	2.9 ± 0.4^b^	15.2 ± 1.4^b^	3.1 ± 0.3^b^

Leaf tissue was sampled from 8-month-old plants of three representative lines. Values represent means from three biological samples ± standard errors. Means are compared column-wise. Values with different letters are significantly different (*p* < 0.0001). *BvLz*_*m*_: maize codon-optimized *bovine lysozyme* (*BvLz*); pUPE:*BvLz*_*m*_: *BvLz*_*m*_ expressed from three promoters, maize *ubiquitin 1*, sugarcane *proline-rich protein* and sugarcane *elongation factor 1α* (pSHEF1α); pUDE:*BvLz*_*m*_: *BvLz*_*m*_ expressed from three promoters, maize *ubiquitin 1*, sugarcane *dirigent16* and pSHEF1α; HF: high fertilization rate; LF: low fertilization rate.

## Conclusions

The genetic/biotechnology tools and resources developed in this study not only expands the utility of sugarcane for large-scale production of recombinant proteins but can be utilized with other monocots and bioenergy feedstocks. Our approach comprises stacking multiple promoters to co-express codon-optimized recombinant genes from different expression vectors using combinatorial transformation methods. This resulted in high recombinant protein yield (up to 11.5% of TSP or 82.5 mg/kg) in transgenic culms, rendering it an attractive biopharming tool for potential commercial uses^69^. We also showed that recombinant BvLz_m_ levels can be maintained stably throughout the growing season and had no negative consequences on sugarcane agronomic performance. Overall, our study provides new knowledge, tools and resources to expand the utility of sugarcane beyond a food crop and bioenergy feedstock to using it as a biofactory for expressing high-value proteins^25^.

## Materials and methods

### Expression vectors

#### Basic vectors

A series of expression vectors were constructed, using a custom synthesized bovine lysozyme (*BvLz*) gene codon-optimized for expression in maize (*BvLz*_*m*_) (444.0 base pairs [bp])^39^ (GenScript, Piscataway, NJ).

The *BvLz*_*m*_ gene was subcloned into pUC57 at *Bam*HI and cloned at the same site into pZero2 (Invitrogen, ThermoFisher Scientific, Waltham, MA), to which the 35ST^34,38,39^ (197.0 bp) was added at the *Pst*I site, resulting in the *BvLz*_*m*_-35ST/pZero2 plasmid.

Three basic *BvLz* expression vectors were generated with the constitutive promoters pUbi^33^, pSHPRP^36^ or pSHEF1α^36^. The first vector, pUbi-*BvLz*_*m*_-35ST/pZero2 was produced by cloning the pUbi fragment (1,977 bp), released from pAHC20 (pUbi:*BAR*/pUC8)^70^ (pUbi minus heat shock element; a 28.0 bp deletion at the 5′ end of pUbi) with *Bam*HI/*Hind*III and filled in, into the filled-in *BvLz*_*m*_-35ST/pZero2. For the other two vectors, the *Sma*I-treated pSHPRP (3,016 bp) and pSHEF1α (1,959 bp) fragments from pSK^+^^36^ were fused to the *Sna*BI/*Bbs*I-treated/filled-in *BvLz*_*m*_-35ST fragment from pUbi-*BvLz*_*m*_-35ST/pZero2 to yield pSHPRP-*BvLz*_*m*_-35ST/pSK^+^ and pSHEF1α-*BvLz*_*m*_-35ST/pSK^+^, respectively.

Two basic *BvLz* expression vectors were generated with the culm-regulated promoters pSHDIR16^35^ or pSCBV21^34^. The pSHDIR16-*BvLz*_*m*_-35ST/pSK^+^ vector was assembled by fusing *BvLz*_*m*_-35ST, excised from *Bam*HI/*Eco*RI-treated *BvLz*_*m*_-35ST/pZero2, to the pSHDIR16 fragment^35^ (2,680 bp) at the same sites in pSK^+^. The pSCBV21-*BvLz*_*m*_-35ST/pGEMT-T Easy vector was produced by cloning *BvLz*_*m*_-35ST, excised from *Bam*HI/*Eco*RI-treated *BvLz*_*m*_-35ST/pZero2, into the *Nco*I-treated/filled-in pSCVB21 (1,816 bp)/pGEM-T Easy^34^.

#### Double terminator vectors

*BvLz* constructs with a double terminator were generated by fusing the NOST (253 bp)^39^ to the 35ST of basic *BvLz* constructs. The pUbi-*BvLz*_*m*_-35STNOST/pZero2 vector was constructed by releasing the NOST from pBI221 (Accession Number AF502128) (Clontech Laboratories, Inc., Mountain View, CA) with *Eco*RI/*Sst*I, filled in and cloned into the *Xho*I-treated/filled-in pUbi-*BvLz*_*m*_-35ST/pZero2. To make pSHPRP-*BvLz*_*m*_-35STNOST/pSK^+^ and pSHEF1α-*BvLz*_*m*_-35STNOST/pSK, the *Sna*BI/*Bbs*I-treated/filled-in *BvLz*_*m*_-35STNOST fragment from pUbi-*BvLz*_*m*_-35STNOST/pZero2 was fused to the *Sma*I-treated pSHPRP/pSK^+^ and pSHEF1α/pSK^+^ vectors, respectively. To generate the pSCBV21-*BvLz*_*m*_-35STNOST/pGEM-T Easy vector, the *Sna*BI/*Bbs*I-treated/filled-in *BvLz*_*m*_-35STNOST fragment from pUbi-*BvLz*_*m*_-35STNOST/pZero2 was cloned into *Nco*I-treated/filled-in pSCVB21/pGEM-T Easy.

#### Vectors with viral untranslated regions

The 3′UTR of SrMV strain H (GenBank Accession Number U57358) (235.0 bp) was custom synthesized as a fusion to *BvLz*_*m*_ in pJI (*BvLz*_*m*_-SrMV 3′UTR/pJI) (ATUM, DNA2.0, Newark, CA). The pUbi-*BvLz*_*m*_-SrMV 3′UTR-35ST/pZero2 vector was assembled by cloning the filled-in SrMV 3′UTR, released from *Eco*RI/*Bgl*II-treated *BvLz*_*m*_-3′SrMV/pJI, into pUbi-*BvLz*_*m*_-35ST/pZero2 at the *Sma*I site. The pSHPRP-*BvLz*_*m*_-SrMV 3′UTR-35ST/pSK^+^ and pSHEF1α-*BvLz*_*m*_-SrMV 3′UTR-35ST/pSK^+^ vectors were generated by fusing the *Sna*BI/*Bbs*I-treated/filled-in *BvLz*_*m*_-SrMV 3′UTR-35ST fragment from the pUbi-*BvLz*_*m*_-SrMV 3′UTR-35ST/pZero2 to pSHPRP/pSK^+^ and pSHEF1α/pSK^+^ at the *Sma*I site, respectively. For construction of pSHDIR16*-BvLz*_*m*_-SrMV 3′UTR-35ST/pSK^+^ vector, SrMV 3′UTR was released from *BvLz*_*m*_-SrMV 3′UTR/pJI by *Eco*RV treatment and cloned into pSHDIR16-*BvLz*_*m*_-35ST/pSK^+^ at the *Eco*RV site.

All DNA cloning steps were carried out as described by Sambrook^71^. Filling in of endonuclease-treated DNA fragments and dephosphorylation of vectors were done using T4 DNA polymerase (NEB BioLabs, Ipswich, MA) and antarctic phosphatase (NEB BioLabs), respectively.

### Sugarcane transformation

Tops of field-grown sugarcane (*Saccharum* spp. hybrids) commercial varieties CP72-1210, CP84-1198, TCP87-3388 and TCP98-4454 were collected during the growing season, and leaf roll discs were prepared for stable transformations as previously described^72^. Briefly, leaf blades and sheaths were removed down to the top visible dewlap leaf, and the upper 20–30 cm portion of shoot (leaf roll culm) was surface sterilized in 70.0% (v/v) ethanol for 20 min. Immature leaf rolls close to the apical meristem were sliced transversely into 1.0 mm thick sections and cultured on MS3 medium (MS medium with 3.0 mg/l of 2,4-dichlorophenoxyacetic acid [2,4-D]) for 30–35 days (for embryogenic calli) or MS0.6 medium (MS with 0.6 mg/l of 2,4-D) for 7–10 days (for embryogenic leaf roll discs). Embryogenic calli and leaf roll discs were preconditioned on MS3- and MS0.6-osmoticum (MS3 or MS0.6 with 0.2 M d-mannitol and 0.2 M d-sorbitol), respectively, for 4 h before and after DNA particle bombardment. DNA bombardment was performed according to Beyene and colleagues^38^. Briefly, tungsten particles (1.1 µm; Bio-Rad Laboratories, Inc.) (1.0 mg) were coated separately with plasmid DNA (1.0 µg) of different constructs at equimolar ratios together with pUbi:*BAR*/pUC8 selectable marker plasmid using calcium chloride (NaCl) (1.0 M) and spermidine (14.0 mM). The DNA particle suspension (containing the selectable marker plasmid with one or more *BvLz*_*m*_ plasmids) (4.0 μl; 0.5 µg DNA per bombardment) was placed at the center of a syringe filter and delivered into tissue with a particle inflow gun using a 26.0-inch Hg vacuum and a 7.0-cm target distance. Bombarded embryogenic calli and leaf roll discs were maintained on MS3 and MS0.6, respectively, for 10 days in the dark at 28 °C for recovery. They were later incubated in the dark at 28 °C on selection medium (MS3 or MS0.6 with bialaphos at 3.0 mg/l) for a total of 2 weeks. Shoot regeneration and root initiation were performed under bialaphos selection as previously described^72^. Rooted plantlets were transferred to potting soil (Sunshine Mix #1; SunGro Horticulture Distribution, Inc., Agawan, MA) in pots and maintained in the greenhouse.

### Transgenic plant screening

#### Integration and size determination of *BvLz*_*m*_ expression cassettes

Integration and size of each *BvLz*_*m*_ expression cassette in the single and multiple stacked promoter:*BvLz*_*m*_ sugarcane lines were determined by Southern blot and PCR analyses, respectively, using genomic DNA isolated according to Tai and Tanksley^73^ from liquid N-ground tissues (3.0 g) collected from young leaves of 3–4 month-old plants. Controls included vector-transformed lines and non-transformed plants (tissue culture-derived).

For Southern blot analysis, genomic DNA (10.0 μg per lane) was treated with *Hind*III endonuclease, electrophoresed on 0.8% (w/v) agarose gels and transferred to nylon membranes (Amersham Hybond-XL, GE Healthcare Bio-Sciences Corp., Piscataway, NJ) in 0.4 M sodium hydroxide^74^. Pre-hybridization, hybridization, washing and detection of DNA gel blots were performed using Church’s buffer^75^. The probe, corresponding to the *BvLz*_*m*_ coding sequence was amplified by PCR from pUbi-*BvLz*_*m*_-35ST/pZero2 using the primer set BvLz-1F (5′-ATGGCGGCCCTGGTGATCCTGGGCT-3′) and BvLz-481R (5′-TCACAGGGTGCAGCCTTCCACG-3′) and labeled with [α-^32^P] dCTP using the Random DNA Labeling kit (Invitrogen, ThermoFisher Scientific).

PCR was performed on a C1000 Touch thermal cycler (Bio-Rad Laboratories, Inc., Hercules, CA) in a total reaction volume of 25.0 µl using 200.0 ng of DNA and Platinum *Taq* DNA polymerase (Invitrogen, ThermoFisher Scientific) according to the manufacturer’s instructions with the following conditions: 94 °C for 4 min, 35 cycles each at 94 °C for 30 s, 49.7–54.4 °C for 30 s, and 72 °C for 6 min. Primers encompassing the entire promoter:*BvLz*_*m*_-terminator cassette (Supplementary Table S4) were designed with Primer 3.0. All PCR amplicons were separated by electrophoresis on 0.7% agarose (w/v) gels stained with ethidium bromide. A “no DNA template” was included as a negative control for PCR.

#### Determination of *BvLz*_*m*_ copy number

*BvLz*_*m*_ copy number in single and multiple stacked promoter:*BvLz*_*m*_ sugarcane lines was estimated by qPCR. qPCR was performed on a CFX384 Real-time PCR Detection System (Bio-Rad Laboratories, Inc.) using i*Taq* Universal SYBR Green Supermix (Bio-Rad Laboratories, Inc.), 0.4 µM of each target specific primer and 1.0 ng of genomic DNA from representative transgenic *BvLz*_*m*_ lines, according to the manufacturer’s instructions. Primers specific to the promoter-*BvLz*_*m*_ gene junction area (Supplementary Table S4) were designed with Primer 3.0 (https://bioinfo.ut.ee/primer3-0.4.0/primer3/). qPCR conditions were as follows: 95.0 °C for 3 min, 39 two-step cycles each at 96.0 °C for 5 s and 57 °C for 30 s, and a final melting curve of 60.0 °C to 95.0 °C for 6 min. The sugarcane anthranilate phosphoribosyltransferase and prolyl 4-hydroxylase genes were used as a reference for single copy genes^76^. qPCR was performed twice in triplicate with two biological replications. PCR efficiency was calculated with LinReg^77^. Results were analyzed and recorded as C_T_ (threshold cycle) values. Copy number of the *BvLz*_*m*_ gene was estimated by qPCR according to Casu et al.^76^ using the formula GCI = Eff^RefC^_T_/Eff^C^_T_, where: GCI = gene copy number index, Eff^RefC^_T_ = PCR efficiency using the reference gene primers to the power of the reference gene C_T_ value for each sample, and Eff^C^_T_ = PCR efficiency using the test gene primers to the power of the test gene C_T_ value generated for each sample.

### Expression analysis of *BvLz*_*m*_

Total RNA was isolated by grinding 1.0 g of young leaves collected from 3–4 month-old plants in liquid N^39,78^. For northern blot analysis, RNA (15.0 μg per lane) was fractionated on 1.6% formaldehyde agarose denaturing gels in HEPES buffer and blotted onto nylon membranes (Amersham Hybond-XL) in 10x SSC^75^. Pre-hybridization, *BvLz*_*m*_ probe labeling, hybridization, washing and detection of RNA gel blots were performed as described for Southern blot analysis.

### Plant growth and treatment conditions

For growth cycle investigations, single-node culm cuttings of 15 single promoter pU:*BvLz*_*m*_ transgenic lines and non-transformed plants were pre-germinated in seedling flats (Supplementary Fig. S1) for 2.5 weeks and transplanted into 37.0-l pots (four pots per line) in commercial growth medium (Sunshine Mix #1). Plants were maintained in a temperature-regulated greenhouse with average day/night temperatures of 32/22 °C and relative humidity of 60–100%. Plants were initially fertilized once per week with a commercial high-phosphorus soluble fertilizer (Peters 8%N-19.8%P-12.5%K; The Scotts Company, Marysville, OH) for 5 weeks and then with a balanced/complete soluble fertilizer (Peters Professional 20–20–20; The Scotts Company) containing N 200.0 g/kg, P 80.0 g/kg, K 166.0 g/kg, Mg 1.0 g/kg, iron 0.5 g/kg, manganese 0.3 g/kg, boron 0.1 g/kg, copper 0.13 g/kg, molybdenum 0.05 g/kg, and zinc 0.25 g/kg.

To assess the impacts of mineral nutrient supply on growth and BvLz_m_ accumulation, plants from four representative triple promoter pUDE:*BvLz*_*m*_ lines and one representative triple promoter pUPE:*BvLz*_*m*_ line were pre-germinated and transplanted into 15.0-l plastic pots containing the same growth medium as described above. All pots were initially fertilized with a high-phosphorus fertilizer (Peters 8%N–19.8%P–12.5%K; Scotts, Marysville, OH; equivalent to 10.0 kg N/ha). After 2 months, pots were randomly assigned into two fertilization treatment groups, namely, high fertility (HF) and low fertility (LF), with four pots per line selected for each group. Non-transformed plants (tissue culture-derived) were included as negative controls. Fertilization treatments were achieved with a complete fertilizer (Peters Professional 20–20–20) containing macro- and micro-nutrients as described above. Plants in the LF group received an additional equivalent of 20.0 kg N/ha whereas HF plants received 50.0 kg N/ha from supplemental fertilization using Peters Professional 20–20–20 (described above). Fertilizer treatments were applied in split doses (twice per week). Transgenic culms were harvested at 2, 6 and 8 months following fertilization, processed, and their BvLz yield was determined by ELISA at the BioSeparation Facility of Texas A&M University’s Biological and Agricultural Engineering Department (College Station, Texas).

### Plant physiological analysis

For inorganic mineral analysis, leaf tissue samples were collected, dried (70 °C for 48 h), ground to pass a 40-μm screen and analyzed for inorganic minerals. Total Kjeldahl N (ammonia and organic N) was determined in digested samples using the EasyChem Plus Analyzer and protocols (Systea Scientific, Chicago, IL), whereas other macronutrients such as P, K and Mg were analyzed using the Optima 7300 DV Inductively Coupled Plasma-Optical Emission Spectrometer (PerkinElmer, Shelton, CT) after partial digestion (hydrolysis) on a HotBlock Digestion System (Environmental Express, Inc., Charleston, SC).

### Total protein extraction

Large-scale extraction and size fractionation of total soluble proteins (TSPs) from culms (300.0 lbs) of *BvLz*_*m*_ transgenic sugarcane were performed at our Pilot Plant Facility mainly as described previously^79^. Bench-scale extraction and purification of BvLz_m_ from extracts of transgenic sugarcane culms (100.0 g), using a single-step hydrophobic interaction chromatography, were performed at our BioSeparation Facility (College Station, Texas) as previously described^30^.

For small-scale extraction of TSP from *BvLz*_*m*_ transgenic sugarcane leaf tissue (200.0 mg) was homogenized in 600.0 µl of sodium acetate buffer (50 mM NaOAc, pH 4.4, 0.1 M NaCl) in 2.0 ml tubes for 30 s at 5,000 rpm with the Precellys 24 homogenizer (MO BIO Laboratories, Carlsbad, CA) using ceramic spherical beads (0.64 cm-diameter). TSP supernatants were collected by centrifugation at 13,000*g* for 25 min at 4 °C.

### Determination of BvLz_m_ accumulation by enzyme activity and enzyme-linked immunosorbent assays

To determine the levels of recombinant BvLz_m_, enzyme activity and enzyme-linked immunosorbent assays (ELISA) were performed on TSP from culm extract juice. Juice was extracted from 1.0 kg of culms of greenhouse grown *BvLz*_*m*_ transgenic plants at 7, 9 and 11 months for the growth cycle experiment and at 2, 6 and 8 months for the fertilization experiment. For enzyme activity determination, culm extract juice was tested for its ability to lyse *Micrococcus lysodeikticus* cells using the standard protocol from Sigma-Aldrich (St. Louis, MO). Rabbit anti-BvLz antibody used in the ELISA was synthesized by Bethyl Laboratories, Inc. (Montgomery, TX) using tobacco-derived BvLz^31^ and further purified through an SP-Sepharose column (GE Healthcare, Piscataway, NJ). ELISA of culm extract juice was performed as previously described^30^. Briefly, a sandwich ELISA consisting of anti-BvLz antibody was used to capture BvLz in juice. Detection was performed using a biotinylated anti-BvLz antibody and horseradish peroxidase-labeled NeutrAvidin (Pierce, ThermoFisher Scientific). The standard curve was generated using BvLz produced in *Pichia pastoris* as in Digan et al.^80^.

### Statistical analysis

Agronomic data were collected from 3 to 4 independent experiments, with 3–4 replicates per experiment and subjected to an analysis of variance (ANOVA) using the General Linear Model procedure of the Statistical Analysis System 9.4 (SAS Institute Inc., Cary, NC). Mean separation was performed using the Student–Newman–Keuls (SNK) test.

## Supplementary information

Supplementary Information.
