# Supplementary material for: Unprecedented enhancement of recombinant protein production in sugarcane culms using a combinatorial promoter stacking system
Source: Sci Rep. 2020 Aug 13;10:13713. doi: 10.1038/s41598-020-70530-z (PMC7426418; doi:10.1038/s41598-020-70530-z)
Supplement: Supplementary file 1 — Supplementary Information. [file 41598_2020_70530_MOESM1_ESM.docx]

***Supplementary Information***

**Unprecedented enhancement of recombinant protein production in sugarcane culms using a combinatorial promoter stacking system**

Mona B. Damaj^1*^, John L. Jifon^1,2^, Susan L. Woodard^3^, Carol Vargas-Bautista^1#^, Georgia O. F. Barros^4^, Joe Molina^1^, Steven G. White^4^, Bassam B. Damaj^5^, Zivko L. Nikolov^4^ and Kranthi K. Mandadi^1,6*^

^1^Texas A&M AgriLife Research and Extension Center, 2415 East US Highway 83, Weslaco, Texas 78596, USA, e-mail mbdamaj@ag.tamu.edu, JLJifon@ag.tamu.edu, joemolina344@gmail.com, carolvargasb@tamu.edu, kkmandadi@tamu.edu

^2^Department of Horticultural Sciences, Texas A&M University, College Station, Texas 77843-2133, USA

**^3^**National Center for Therapeutics Manufacturing, 100 Discovery Drive, Texas A&M University, College Station, Texas 77843-4482, USA, e-mail woodard.sue@gmail.com

**^4^**BioSeparation Laboratory, Biological and Agricultural Engineering Department, College Station, Texas 77843-2117, USA, e-mail georgiabarros@yahoo.com.br, sgwhite3852@gmail.com, znikolov@tamu.edu

^5^Innovus Pharmaceuticals, Inc., 8845 Rehco Road, San Diego, California 92121, USA, e-mail bdamaj@innovuspharma.com

^6^Department of Plant Pathology and Microbiology, Texas A&M University, College Station, Texas 77843-2132, USA

^#^Current address: College of Medicine, 8447 Riverside Parkway, Texas A&M University, Bryan, Texas 77807, USA

***Corresponding authors:** kkmandadi@tamu.edu and mbdamaj@ag.tamu.edu

| *Bovine lysozyme (BvLz_m_)* expressing line | BvLz_m_ yield  (mg/kg culm mass) | | Pearson’s correlation coefficient (R) |
| --- | --- | --- | --- |
|  | **Enzyme-linked immunosorbent assay** | **Enzyme activity assay** |  |
| *pU:BvLz_m_-single terminator (35ST)* | | | |
| 3 | 0.18 ± 0.01 | 0.19 ± 0.02 | 0.9777 |
| 13 | 0.20 ± 0.02 | 0.25 ± 0.01 | 0.9286 |
| 19 | 0.15 ± 0.01 | 0.17 ± 0.02 | 0.9449 |
| *pUD:BvLz_m_-single terminator (35ST)* | | | |
| 18 | 0.50 ± 0.01 | 0.53 ± 0.03 | 0.9631 |
| 42 | 0.60 ± 0.03 | 0.63 ± 0.03 | 0.8109 |
| 91 | 0.50 ± 0.04 | 0.57 ± 0.02 | 0.9558 |
| *pUPE:BvLz_m_-3’UTR-35ST* | | | |
| 11 | 1.4 ± 0.1 | 1.6 ± 0.2 | 0.9790 |
| 22 | 2.0 ± 0.2 | 2.5 ± 0.2 | 0.9830 |
| 24 | 3.5 ± 0.3 | 4.0 ± 0.3 | 0.8510 |
| *pUDE:BvLz_m_-3’UTR-35ST* | | | |
| 1 | 2.3 ± 0.1 | 2.8 ± 0.3 | 0.8825 |
| 10 | 3.7 ± 0.3 | 3.9 ± 0.7 | 0.9820 |
| 20 | 5.3 ± 0.3 | 5.5 ± 0.8 | 0.8386 |
| *pUPBE:BvLz_m_-3’UTR-35ST* | | | |
| 1 | 6.3 ± 0.4 | 6.5 ± 0.9 | 0.8743 |
| 4 | 6.0 ± 0.3 | 6.3 ± 0.3 | 0.8331 |
| 15 | 6.0 ± 0.2 | 6.5 ± 0.7 | 0.8556 |
| *pUPBE:BvLz_m_-35STNOST* | | | |
| 1 | 6.7 ± 0.3 | 7.5 ± 0.4 | 0.9475 |
| 2 | 10.0 ± 0.7 | 11.1 ± 0.5 | 0.9377 |
| 4 | 8.3 ± 0.4 | 8.7 ± 0.9 | 0.8910 |
| *pUDE:BvLz_m_ + pP:BvLz_m_ + pB:BvLz_m_* | | | |
| 1 | 11.8 ± 1.4 | 12.5 ± 1.0 | 0.8660 |
| 5 | 14.6 ± 2.3 | 15.6 ± 1.5 | 0.9658 |
| 12 | 28.6 ± 3.4 | 31.1 ± 2.4 | 0.8727 |

**Supplementary Table S1.** Recombinant bovine lysozyme (BvLz_m_) yield of representative sugarcane transgenic lines as determined by enzyme activity and enzyme-linked immunosorbent assays. Data represent five biological replications with standard errors from three assays. *BvLz_m_*: maize codon-optimized *BvLz*; pU: maize *ubiquitin 1* promoter; pUD:*BvLz_m_*: *BvLz_m_* expressed from two promoters, maize *ubiquitin 1* and sugarcane *dirigent16* (pD); pUPE:*BvLz_m_*: *BvLz_m_* expressed from three promoters, pU*,* sugarcane *proline-rich protein* (pP) and sugarcane *elongation factor 1α* (pE); pUDE:*BvLz_m_*: *BvLz_m_* expressed from three promoters, pU*,* pD and pE; pUPBE:*BvLz_m_*: *BvLz_m_* expressed from four promoters, pU, pP, pE and *Sugarcane bacilliform virus*; 3’UTR: 3’ untranslated region of *Sorghum mosaic virus*; 35ST: *Cauliflower mosaic virus* 35S terminator; NOST: *Agrobacterium tumefaciens* nopaline synthase terminator. The BvLz_m_ yield is determined in juice extract of culms (one kg of culm).

| Single promoter:*bovine lysozyme* (*BvLz_m_*) expressing line | BvLz_m_ yield as determined by ELISA | |
| --- | --- | --- |
|  | **BvLz_m_ (mg/kg culm mass)** | **TSP (%)** |
| pUbi:*BvLz_m_*-single terminator lines  6.7%  40.0%  53.3% | 0.08-0.4 (range)  0.08-0.1  0.12-0.18  0.2-0.4 | 0.01-0.06 (range)  0.01-0.015  0.017-0.027  0.03-0.06 |
| pSHEF1α:*BvLz_m_*-single terminator lines  54.7%  45.3% | 0.1-0.4 (range)  0.1-0.25  0.3-0.4 | 0.1-0.06 (range)  0.01-0.038  0.04-0.06 |
| pSHPRP:*BvLz_m_*-single terminator lines  27.6%  72.4% | 0.2-0.4 (range)  0.2-0.25  0.3-0.4 | 0.03-0.06 (range)  0.03-0.038  0.04-0.06 |
| pSHDIR16:*BvLz_m_*-single terminator lines  62.8%  37.2% | 0.3-0.56 (range)  0.3-0.37  0.4-0.56 | 0.04-0.08 (range)  0.04-0.056  0.05-0.07 |
| pSCBV21:*BvLz_m_*-single terminator lines  42.9%  57.1% | 0.4-0.56 (range)  0.4-0.48  0.5-0.56 | 0.05-0.08 (range)  0.05-0.067  0.07-0.08 |

**Supplementary Table S2.** Recombinant bovine lysozyme (BvLz_m_) yield of representative single promoter:*BvLz_m_* expressing sugarcane lines. The percentage (%) of plants (11-12-month-old) carrying the single promoter:*BvLz_m_* is presented under each line category. *BvLz_m_*: maize codon-optimized *BvLz*; pUbi: maize *ubiquitin 1* promoter; pSHEF1α: sugarcane *elongation factor 1α* promoter; pSHPRP: promoter for sugarcane *proline-rich protein*; pSHDIR16: sugarcane *dirigent16* promoter; pSCBV21: *Sugarcane bacilliform virus* promoter; ELISA: enzyme-linked immunosorbent assay; TSP: total soluble protein.

| *Bovine lysozyme (BvLz_m_)* expressing line | BvLz_m_ yield (mg/kg culm mass) | | | | |
| --- | --- | --- | --- | --- | --- |
|  | **V0** | **V1**  **(Second year)** | **V2**  **(Third year)** | **V3**  **(Fifth year)** | V4  (Seventh year) |
| Triple promoter:*BvLz_m_* expressing lines | | | | | |
| - pUPE:*BvLz_m_* 32 | 2.7 ± 0.1 | 2.7 ± 0.4 | 2.5 ± 0.3 | 2.3 ± 0.2 | 2.4 ± 0.1 |
| - pUDE:*BvLz_m_*  18 | 5.1 ± 0.3 | 4.9 ± 0.8 | 4.7 ± 0.7 | 4.6 ± 0.5 | 4.4 ± 0.3 |
| 19 | 4.6 ± 0.2 | 4.5 ± 0.2 | 4.3 ± 0.3 | 4.2 ± 0.4 | 4.0 ± 0.3 |
| 44 | 2.9 ± 0.1 | 2.9 ± 0.4 | 2.8 ± 0.4 | 2.7 ± 0.3 | 2.7 ± 0.2 |
| 54 | 6.0 ± 0.3 | 5.8 ± 0.9 | 5.7 ± 0.6 | 5.5 ± 0.5 | 5.3 ± 0.4 |

**Supplementary Table S3.** Recombinant bovine lysozyme (BvLz_m_) yield of representative *BvLz_m_* transgenic sugarcane lines after multiple vegetative generations. The BvLz_m_ yield is indicated as determined by enzyme-linked immunosorbent assay in juice extract of culms over four vegetative generations (V) (reproduced by culm cuttings from *BvLz_m_* expressing plants). Data represent five biological replications with standard errors from three assays. *BvLz_m_*: maize codon-optimized *BvLz*; pUPE:*BvLz_m_*: *BvLz_m_* expressed from three promoters, maize *ubiquitin 1* (pUbi1; pU), sugarcane proline-rich protein (pP) and sugarcane elongation factor 1α (pSHEF1α; pE); pUDE:*BvLz_m_*: *BvLz_m_* expressed from three promoters, pUbi, sugarcane *dirigent16* (pD) and pSHEF1α.

| Primer | Primer sequence (5’-3’) |
| --- | --- |
| *For determination of promoter:BvLz_m_ cassette integration* | |
| 1. pUbi-F  2. pSHDIR16-F  3. pSCBV21-F  4. pSHEF1α-F  5. pSHPRP-F  6. NOST-R  7. 35ST-R | TGTGCATGTGTTCTCCTTTTT  GCCTCCTCCTACAGCTCCTT  CAGATGCTTGTGCAACTGGT  CACTTGTTCCCTTGCTGGTT  AGCCGATGAGTTGGGTATTG  ATTGCCAAATGTTTGAACGA  GCTCAACACATGAGCGAAAC |
| *For determination of BvLz_m_ copy number* | |
| 1. pUbi-*BvLz_m_*-F  2. pSHDIR16-*BvLz_m_*-F  3. pSCBV21-*BvLz_m_*-F  4. pSHEF1α-*BvLz_m_*-F  5. pSHPRP-*BvLz_m_*-F  *6. BvLz_m_*-Promoter-R | TTTAGCCCTGCCTTCATACG  TGCACTGCAGCCCTCTATAA  GTGTGTGTTCCCTCTGCCTA  GTGTAGCGTTTCCCTTCAGC  GGCAGAGAGCTAGCAACACA  CTAGCCAGTTCGGACCTTTC |

**Supplementary Table S4.** Primers used in this study*. BvLz_m_*: maize codon-optimized *bovine lysozyme* (*BvLz*); pUbi: maize *ubiquitin 1* promoter; pSHDIR16: sugarcane *dirigent16* promoter; pSCBV21: *Sugarcane bacilliform virus* promoter; pSEF1α: sugarcane *elongation factor 1α* promoter; and pSHPRP: promoter for sugarcane *proline-rich protein.* F: forward primer and R: reverse primer.


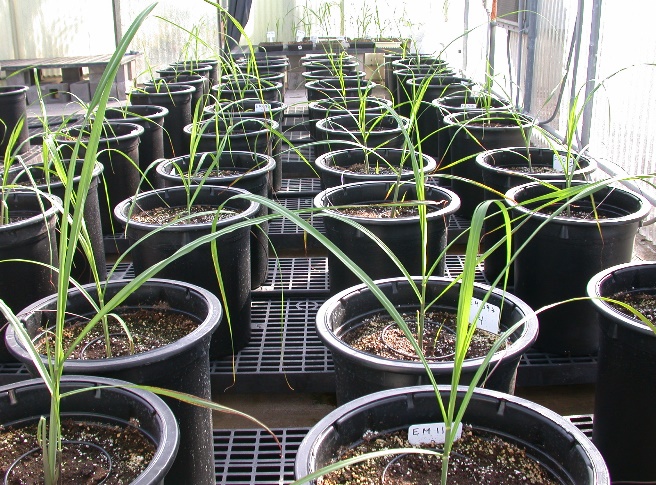

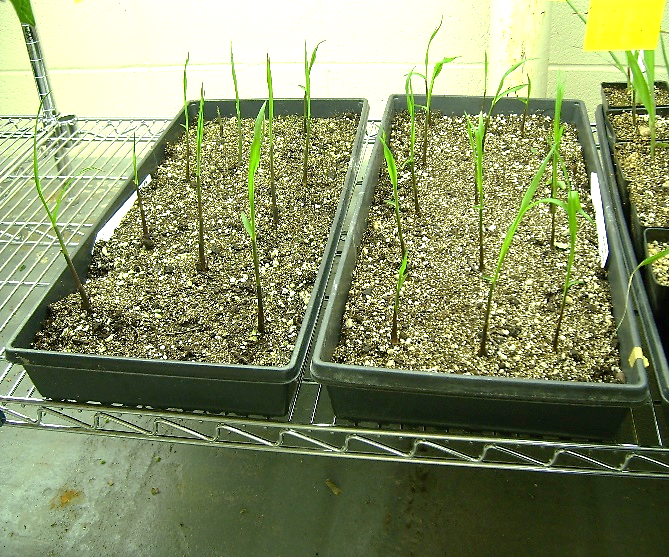

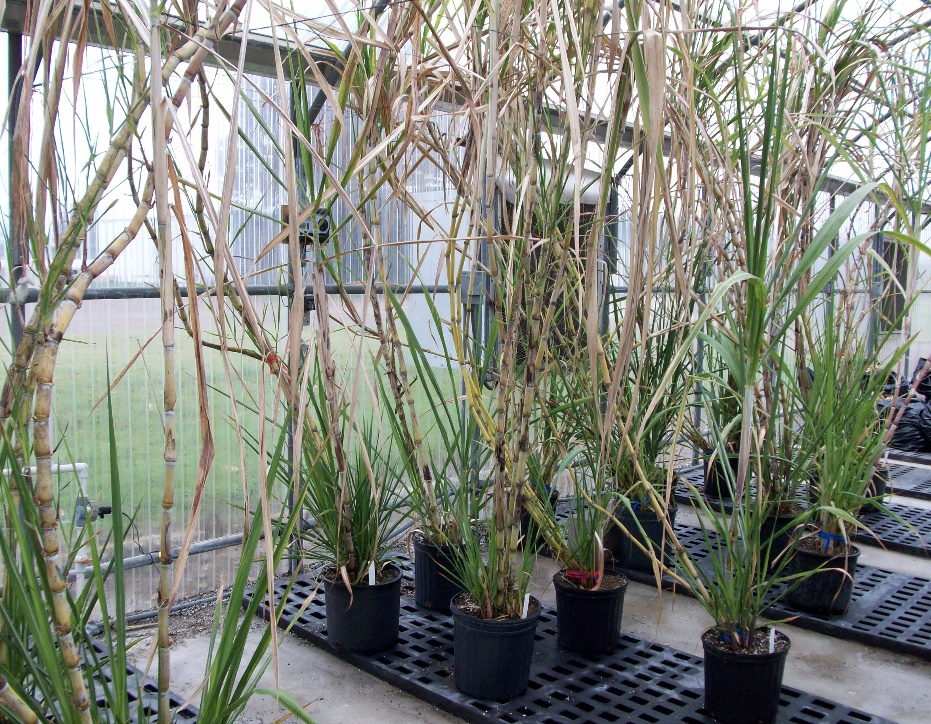


**NT**

**BvLz**

**BvLz**

**NT**

**BvLz**

**WT**

**BvLz**

**a**

**b**

**c**

**Supplementary Figure S1.** Qualitative phenotype of the triple promoter:*bovine lysozyme* expressing sugarcane lines. (**a**) Sprouted seedlings started from single-node culm pieces. (**b**) Plants at seedling stage (2-week-old). (**c**) Mature plants (one-year-old); culms after stripped of their leaves and ready for harvesting, grinding and large-scale protein extraction. BvLz (bovine lysozyme): *BvLz* transgenic plants; NT: non-transformed plants.

**b**


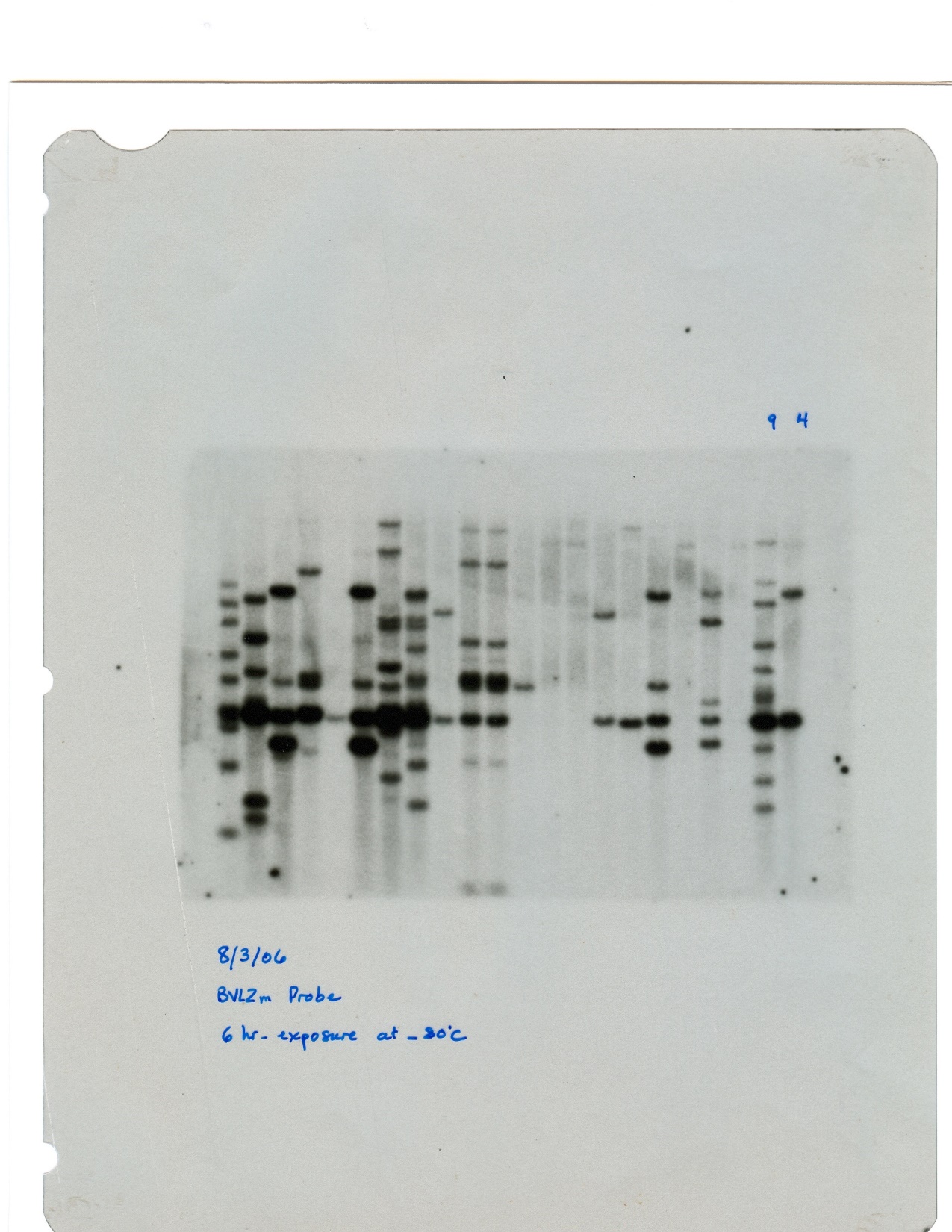


**4**

**9**

**18**

**10**

**11**

**13**

**19**

**3**

**30**

**67**

**108**

**114 116 123**

**1**

**10**

**20**

**16**

**18**

**42**

**91**

**5**

**a**

**11.50**

**2.84**

**2.14**

**1.70**

**1.16**

**0.81**

**5.08**

**Kb**

**11.50**

**2.84**

**2.14**

**1.70**

**1.16**

**0.81**

**5.08**

**Kb**

**1**

**9**

**15**

**17**

**20**

**21**

**18**

**19**

**32**

**33**

**23**

**44**

**54**

**2**

**4**

**10**

**7**

**1**

**3**

**1D**

**1E**


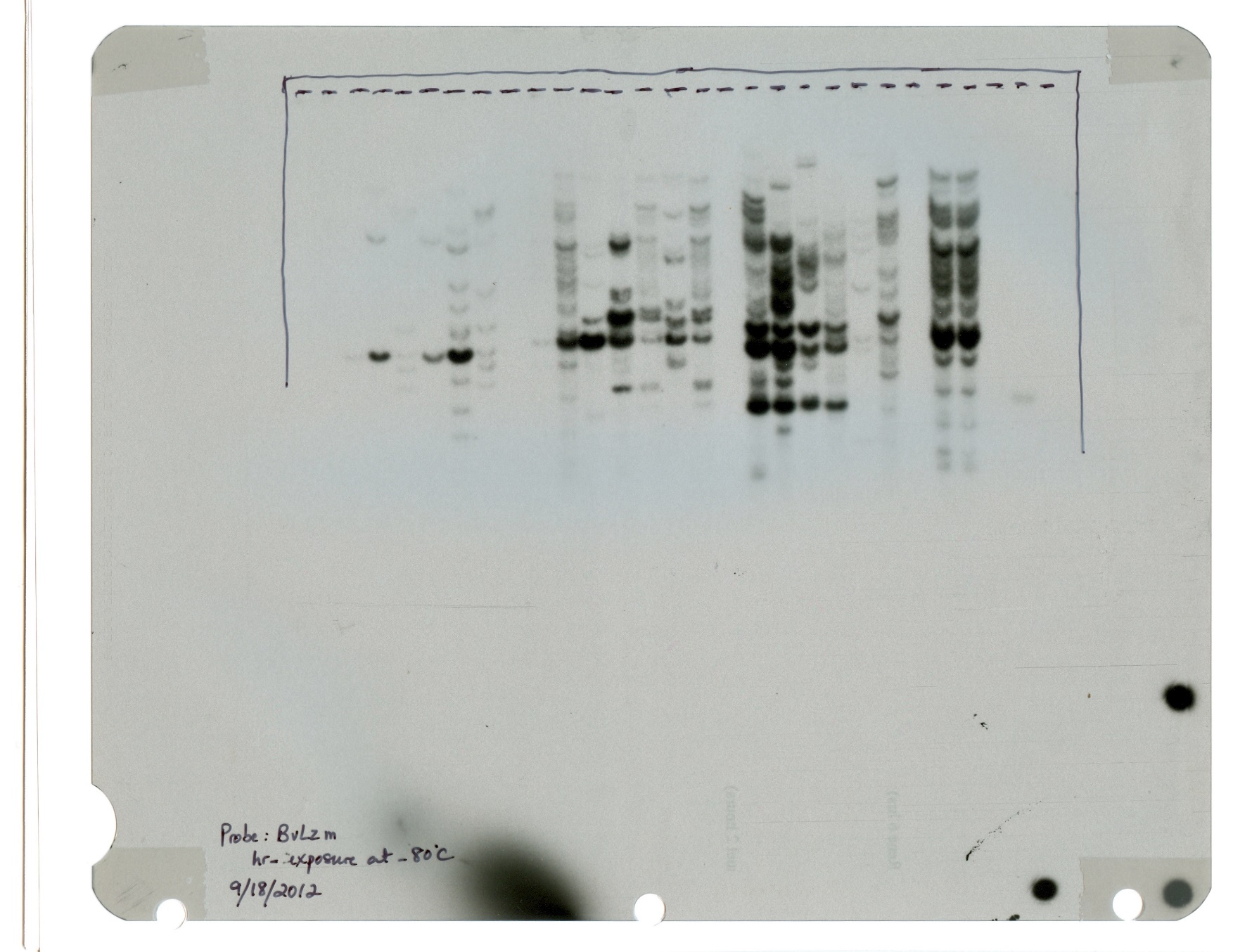


**pUD:*BvLz_m_* line**

**pU:*BvLz_m_* line**

**pUDE:**

***BvLz_m_* line**

**5.08**

**pUDEPB:**

***BvLz_m_* line**

**pUDE:*BvLz_m_* line**

**pU:*BvLz_m_* line**

**pUPBE:**

***BvLz_m_* line**

**Supplementary Figure S2.** (**a**) and (**b**) Full-length uncropped DNA gel blot autoradiograms used to prepare Southern blots in Figure 2a. (**a**) Single promoter pU:*BvLz_m_* lines 4, 9, 18, 10, 11, 13, 19, 3, 30, 67, 108, 114, 116, 123; triple promoter pUDE:*BvLz_m_* lines 1, 10 & 20; double promoter pUD:*BvLz_m_* lines 16, 18, 42, 91 & 50. (**b**) Single promoter pU:*BvLz_m_* lines 1, 9, 15, 17, 20 & 21; triple promoter pUDE:*BvLz_m_* lines 18, 19, 32, 33, 23, 44 & 54; quadruple promoter pUPBE:*BvLz_m_* lines 2, 4, 10, 7, 1 & 3; stacked five promoter pUDEPB: *BvLz_m_* lines 1D & 1E. *BvLz_m_*: maize codon-optimized *BvLz*; U: maize *ubiquitin 1* promoter; D: sugarcane *dirigent16*; E: sugarcane *elongation factor 1α*; P: sugarcane *proline-rich protein*; B: *Sugarcane bacilliform virus*.


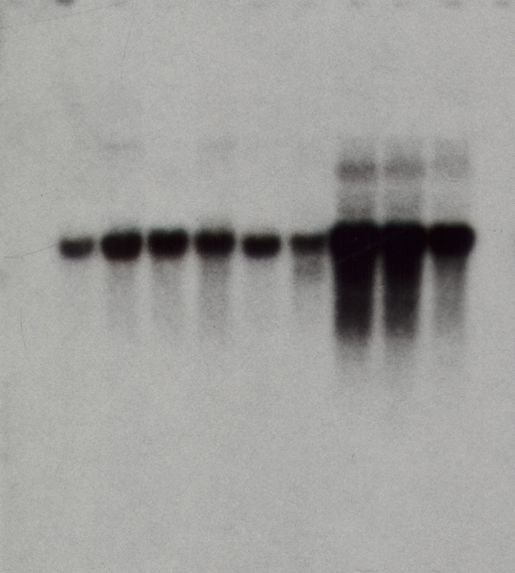


**a**

**1 2 3 4 5 6 7 8 9 10 11 12 13 14**

**1 2 3 4 5 6 7 8 9**


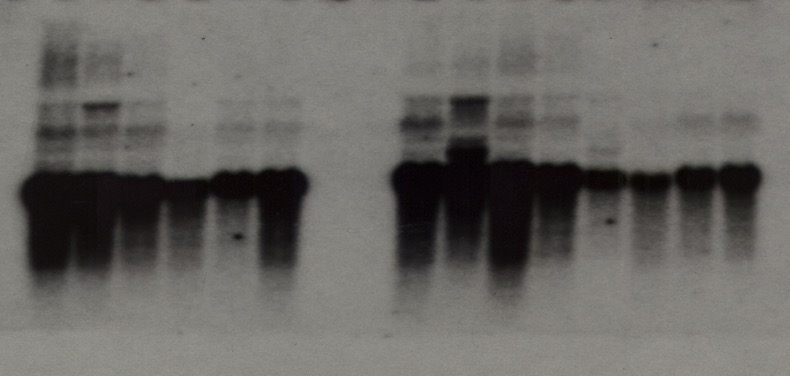


***BvL*z*_m_* transcript**

***BvL*z*_m_* transcript**

**b**

**Supplementary Figure S3.** (**a**) and (**b**) Full-length uncropped RNA gel blot autoradiograms used to prepare northern blots in Figure 2b. (**a**) Lanes 1 and 5-6: single promoter pU:*BvLz_m_* lines 3, 19 & 13; lanes 2-4: double promoter pUD:*BvLz_m_* lines 18, 42 & 91; lanes 7-9: triple promoter pUDE:*BvLz_m_* lines 1, 10 & 20. (**b**) Lanes 1-3 and 7-10: quadruple promoter pUPBE:*BvLz_m_* lines 1, 3, 5, 2, 4, 10 & 7; lanes 11-14: double promoter pUD:*BvLz_m_* lines 16, 17, 21 & 50. *BvLz_m_*: maize codon-optimized *BvLz*; U: maize *ubiquitin 1* promoter; D: sugarcane *dirigent16*; E: sugarcane *elongation factor 1α*; P: sugarcane *proline-rich protein*; B: *Sugarcane bacilliform virus*.

**L 1 2 3 4 5 6 7 8 9 10 11 12**


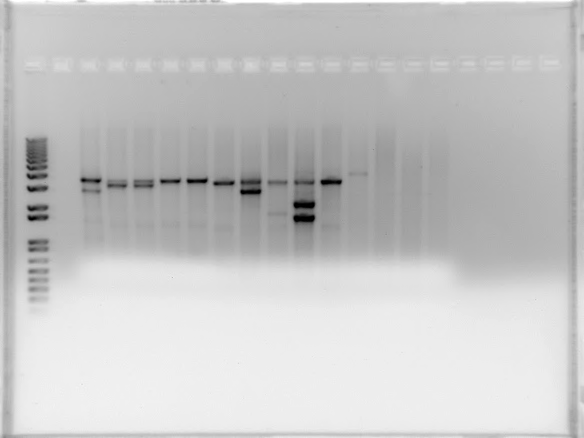


**L 1 2 3 4 5 6 7 8 9 10 11 12 13 14 15 16 17**


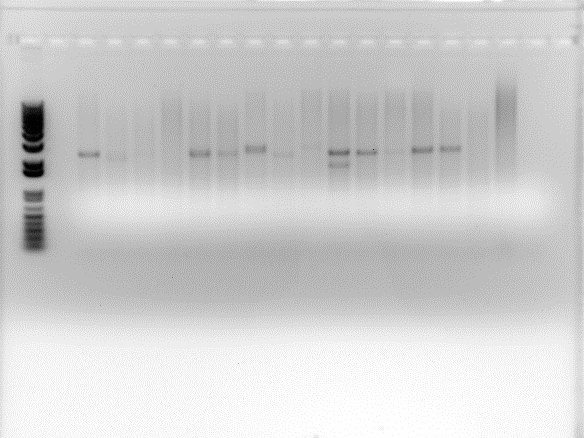

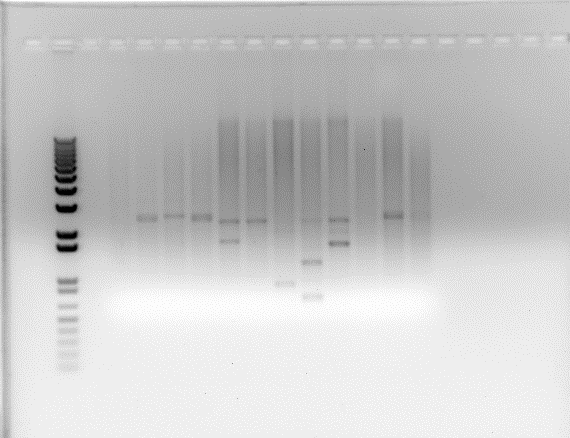


**a**

**b**

**0.1**

**1.0**

**1.65**

**2.0**

**3.0**

**4.0**

**5.0**

**12.0**

**3.0**

**4.0**

**1.65**

**2.0**

**0.1**

**5.0**

**12.0**

**1.0**

**Kb**

**Kb**

**L 1 2 3 4 5 6 7 8 9 10 11 12 13 14 15 16 17**

**(2)**

**(1)**

**Kb**

**12.0**

**4.0**

**5.0**

**1.65**

**2.0**

**3.0**

**1.0**

**0.1**

**Supplementary Figure S4.** (**a**) and (**b**) Full-length uncropped gels of PCR products used to prepare Figures 3a and 3b, respectively. (**a**) Detection of pUbi, *BvLz_m_*, 3’UTR, 35ST and NOST with pUbi-F/35ST-R (2.62 kilobase pairs [kb] or 2.85 kb fragment) and pUbi-F/NOST-R (2.87 kb fragment) primer sets. (1) Lanes 1, 3, 5 & 6: pUDE:*BvLz_m_-*3’UTR-35ST lines 23, 33, 10 & 20; lane 2: pU:*BvLz_m_-*35ST line 13; lanes 4, 7 & 9: pUPBE:*BvLz_m_-*35STNOST lines 2, 4 & 10; lane 8: pUD:*BvLz_m_-*35ST line 91; lanes 10-11: pUPBE:*BvLz_m_-*3’UTR-35ST lines 1 & 4; lanes 12-14: pUPE:*BvLz_m_-*3’UTR-35ST lines 11, 22 & 24; lanes 15-16: Vector-transformed (VT) line, non-transformed (NT) plant & no DNA template. (2) Lanes 1, 6-8: pU:*BvLz_m_-*35ST lines 22, 3, 23 & 13; lane 2: pUPBE:*BvLz_m_-*3’UTR-35ST line 15; lane 3: pUPE:*BvLz_m_-*3’UTR-35ST line 11; lanes 4, 11 & 12: pUDE:*BvLz_m_-*3’UTR-35ST lines 1, 18 & 19; lanes 5, 9 & 10: pUD:*BvLz_m_-*35ST lines 18, 42 & 51. (**b**) Detection of pSHDIR16, *BvLz_m_*, 3’UTR, 35ST and NOST with pSHDIR16-F/35ST-R (3.32 kb fragment) and pSHDIR16-F/NOST-R (3.90 kb fragment) primer sets. Lanes 1-5 & 11: pUDE:*BvLz_m_-*3’UTR-35ST lines 1, 19, 23, 10, 20 & 33; lanes 6-10: pUD:*BvLz_m_-*35ST lines 18, 42, 91, 16 & 50; lane 11: pUPE:*BvLz_m_-*3’UTR-35ST line 11; lane 12: pUPBE:*BvLz_m_-*3’UTR-35ST line 1; lane 13: pUP:*BvLz_m_-*35STNOST line 4; lanes 15-17: VT, NT & no DNA template. L: 1 kb Plus DNA ladder (ThermoFisher Scientific).


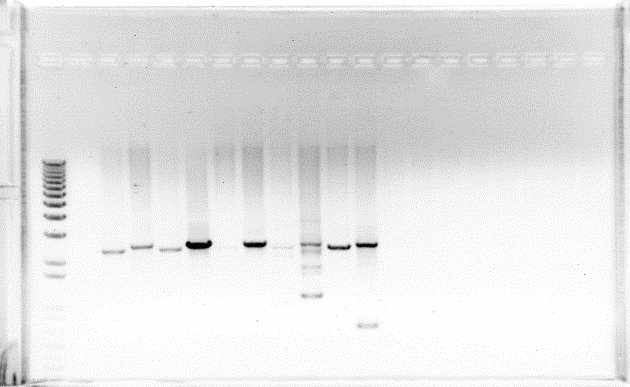


**b**

**a**

**(1)**

**(2)**

**L 1 2 3 4 5 6 7 8 9 10 11 12 13 14 15 16**


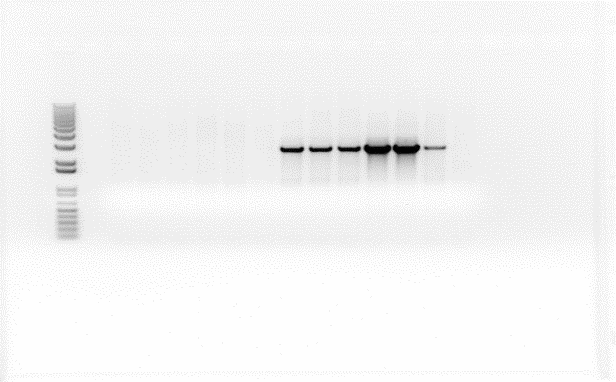


**L 1 2 3 4 5 6 7 8 9 10 11 12 13 14 15**


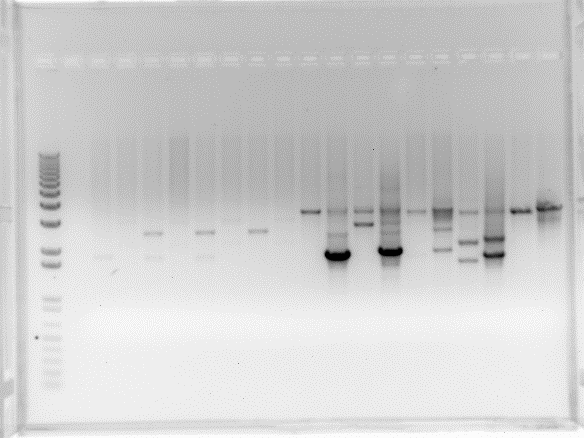


**L 1 2 3 4 5 6 7 8 9 10 11 12 13 14 15 16 17 18**

**(1)**

**(2)**


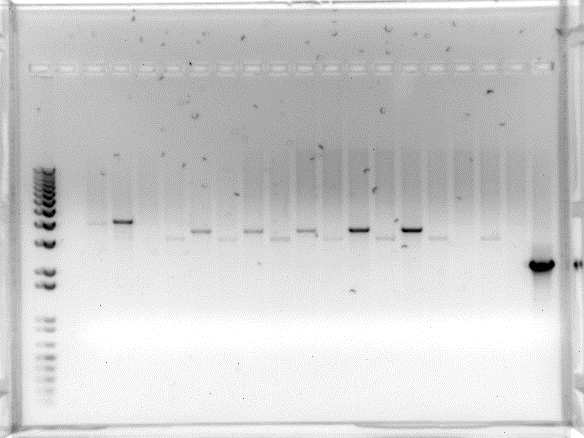


**L 1 2 3 4 5 6 7 8 9 10 11 12 13 14 15 16 17 18**

**2.0**

**4.0**

**0.1**

**12.0**

**1.0**

**Kb**

**1.65**

**3.0**

**5.0**

**2.0**

**0.1**

**1.0**

**4.0**

**12.0**

**Kb**

**5.0**

**1.65**

**3.0**

**12.0**

**1.0**

**2.0**

**4.0**

**Kb**

**12.0**

**0.3**

**1.0**

**4.0**

**5.0**

**Kb**

**3.0**

**1.65**

**0.1**

**2.0**

**3.0**

**1.65**

**5.0**

**Supplementary Figure S5.** (**a**) and (**b**) Uncropped PCR product gel images used to prepare Figures 3c and 3d, respectively. (**a**) and (**b**) Detection of pSHPRP, *BvLz_m_*, 3’UTR, 35ST and NOST using pSHPRP-F/35ST-R (3.65 kb fragment) and pSHPRP-F/NOST-R (3.90 kb fragment) primer sets. (**a**) (1) Lanes 1-2: pUPBE:*BvLz_m_-*35STNOST lines 1 & 2; lane 3: Non-transformed (NT) plant; lanes 4-6: pUPE:*BvLz_m_-*3’UTR-35ST lines 1A, 11, 1 & 1C; lanes 7-18: pUPBE:*BvLz_m_-*3’UTR-35ST lines 1, 1C, 2A, 2D, 5A, 5F, 7A, 7B, 10D, 10F & 1F. (**a**) (2) Lanes 9, 13,17 & 18: pUPE:*BvLz_m_-*3’UTR-35ST lines 22, 1, 2 & 24; lanes 10-12 & 14-16: pUPBE:*BvLz_m_-*3’UTR-35ST lines 4, 15, 17, 6, 7 & 8. (**b**) (1) Lanes 13-15: pU:*BvLz_m_-*35ST line 13, pUD:*BvLz_m_-*35ST line 18 & pUDE:*BvLz_m_-*35ST line 1. (**b**) (2) Lanes 14-16: Vector-transformed (VT) line, NT plant & no DNA template. (**a)** and (**b**) Detection of pSHEF1α, *BvLz_m_*, 3’UTR, 35ST and NOST with pSHEF1α-F/35ST-R (2.57 kb fragment) & pSHEF1α-F/NOST-R (2.82 kb fragment) primer sets. (**a**) (2) Lanes 1, 3, 5 & 7 and lanes 2, 4, 6 & 8: pUPBE:*BvLz_m_-*35STNOST lines 3, 5, 7 & 9 with pSHEF1α-F/35ST-R & pSHEF1α-F/NOST-R primer sets, respectively. (**b**) (1) Lanes 1, 2 & 5: pU:*BvLz_m_-*35ST lines 13, 19 & 3; lanes 3, 4 & 6: pUD:*BvLz_m_-*35ST lines 42, 91 & 18; lanes 7 & 11: pUDE:*BvLz_m_-*3’UTR-35ST lines 1 & 10; lanes 8 &10: pUPE:*BvLz_m_-*3’UTR-35ST lines 11 & 22; lane 9 & 12: pUPBE:*BvLz_m_-*3’UTR-35ST lines 1 & 15. (**b**) (2) Lanes 1, 3, 5, 7 & 9 and lanes 2, 4, 6, 8 & 10: pUPBE:*BvLz_m_-*35STNOST lines 1, 2, 7, 4 & 10 with pSHEF1α-F/35ST-R & pSHEF1α-F/NOST-R primer sets, respectively. Lanes 11-13: VT, NT & no DNA template. L: 1 kb Plus DNA ladder (ThermoFisher Scientific).


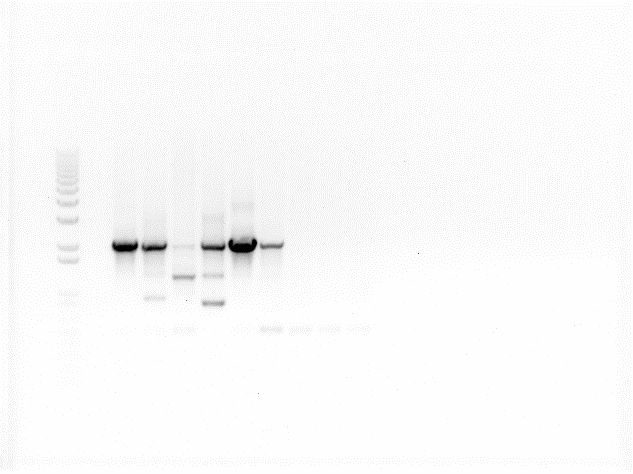


**a**

**L 1 2 3 4 5 6 7 8 9 10**


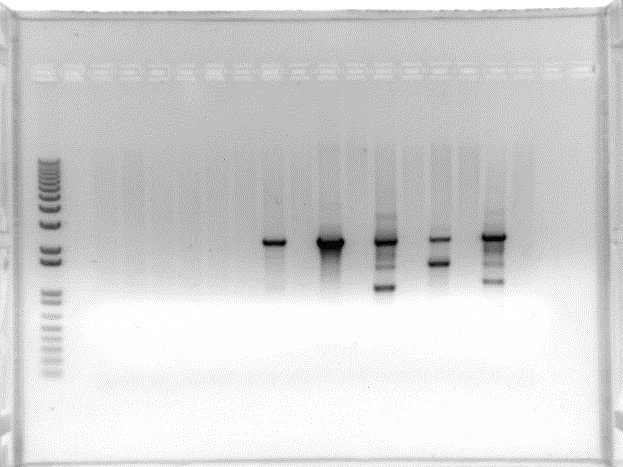


**L 1 2 3 4 5 6 7 8 9 10 11 12 13 14 15 16**

**b**

**Kb**

**12.0**

**2.0**

**3.0**

**4.0**

**5.0**

**1.0**

**1.65**

**0.1**

**Kb**

**12.0**

**3.0**

**4.0**

**5.0**

**1.0**

**1.65**

**2.0**

**0.1**

**Supplementary Figure S6.** (**a**) and (**b**) Uncropped PCR product gel images used to prepare Figure 3e. (**a**) and (**b**) Detection of pSCBV21, *BvLz_m_*, 3’UTR, 35ST and NOST using pSCBV21-F/35ST-R (2.21 kb fragment) and pSCBV21-F/NOST-R (2.46 kb fragment) primer sets. (**a**) Lanes 1-6: pUPBE:*BvLz_m_-*3’UTR-35ST lines 2, 7, 11, 15, 4 & 1; lane 7: pUPE:*BvLz_m_-*3’UTR-35ST line 11; lane 8: pUDE:*BvLz_m_-*3’UTR-35ST line 1; lane 9: pUD:*BvLz_m_*-35ST line 18; lane 10: pU:*BvLz_m_-*35ST line 3. (**b**) Lane 1: pU:*BvLz_m_-*35ST line 13; lane 2: pUD:*BvLz_m_*-35ST line 42; lane 3: pUDE:*BvLz_m_-*3’UTR-35ST line 10; Lane 4-6: Vector-transformed line, NT plant & no DNA template; lane 7-16: pUPBE:*BvLz_m_-*35STNOST lines 10, 2E, 4, 9C, 2, 1D, 1, 8E, 7 & 7E. L: 1 kb Plus DNA ladder (ThermoFisher Scientific).
